# Regulation of the tenogenic gene expression in equine tenocyte-derived induced pluripotent stem cells by mechanical loading and Mohawk

**DOI:** 10.1016/j.scr.2019.101489

**Published:** 2019-06-27

**Authors:** Feikun Yang, Aiwu Zhang, Dean W. Richardson

**Affiliations:** Department of Clinic Studies at New Bolton Center, University of Pennsylvania, 382 West Street Road, Kennett Square, PA 19348, United States of America

**Keywords:** Tenogenic differentiation, Induced pluripotent stem cell (iPSC), Bone marrow mesenchymal stem cell (BMSC), Mechanical loading, Mohawk

## Abstract

Cell-based therapeutic strategies afford major potential advantages in the repair of injured tendons. Generation of induced pluripotent stem cells (iPSCs) expands cell sources for “regenerative” therapy. However, its application in tendon repair is still limited and the effects remain unclear. In this study, equine tenocyte-derived iPSCs (teno-iPSCs) were generated by expressing four Yamanaka factors. Compared to parental tenocytes and bone marrow derived mesenchymal stem cells (BMSCs), the transcriptional activities of lineage-specific genes, including *Mkx*, *Col1A2*, *Col14*, *DCN*, *ELN*, *FMOD,* and *TNC*, were highly repressed in the resulting teno-iPSCs. Exposure to cyclic uniaxial mechanical loading increased the expression of *Scx*, *Egr1*, *Col1A2*, *DCN,* and *TNC* in teno-iPSCs and the expression of *Scx*, *Egr1*, *DCN,* and *TNC* in BMSCs. Reintroduction of tenogenic transcription factor Mohawk (*Mkx)* upregulated the expression of *DCN* in teno-iPSCs and the expression of *Scx*, *Col14,* and *FMOD* in BMSCs. Mechanical loading combined with ectopic expression of equine *Mkx* further enhanced the expression of *Egr1*, *Col1A2*, *DCN,* and *TNC* in teno-iPSCs and the expression of *Scx*, *Egr1,* and *TNC* in BMSCs. These data suggest that the repressed lineage-specific genes in the teno-iPSCs can be re-activated by mechanical loading and ectopic expression of *Mkx*. Our findings offer new insights into the application of iPSCs for basic and clinic research in tendon repair.

## 1. Introduction

Tendon is a unique form of connective tissue that transmits the force from muscle to bone, thus allowing joint movement efficiently (Nourissat et al., 2015). Tendon injuries are common diseases in the musculoskeletal system, affecting both human and veterinary species (Docheva et al., 2015). Due to the limited capacity for regeneration, the natural healing process for damaged tendon is slow and often insufficient (Ho et al., 2014).

Cell-based tissue engineering approaches show great promise in tendon therapy, and a large number of cell populations, including tenocytes, mesenchymal stem cells (MSCs), embryonic stem cells (ESCs) and tendon progenitor/stem cells (TPSCs), have been proposed for tendon repair and regeneration under various conditions (Gaspar et al., 2015). However, none of the tested cells is perfect. For example, although MSCs have been extensively studied for tendon repair, their proliferation and differentiation potential are affected by the age of their origin and the *in vitro* culture conditions (Beane et al., 2014). Moreover, the limited passage number and increasing cellular senescence during passaging also hinder their application in regenerative. medicine (Turinetto et al., 2016). TPSCs show the capacity to form a tendon-like tissue *in vitro* and *in vivo* (Bi et al., 2007; Tan et al., 2012), but they are also prone to lose their phenotype along with continuous passages (Webb et al., 2016). iPSCs derived from differentiated somatic cells expand the cell sources and hold high potential for cell therapy and tissue engineering. However, their application in tendon regeneration is still very limited and the effects remain debatable (Sun et al., 2015; Zhang et al., 2015; Czaplewski et al., 2014; Xu et al., 2013; Bavin et al., 2015). While Xu et al. reported that human iPSC-derived neural crest stem cells could promote tendon repair in a rat patellar tendon window defect model(Xu et al., 2013), the study from Bavin et al. showed that, compared to embryonic stem cells, equine fetal fibroblast-derived iPSCs had a reduced tendon differentiation capacity (Bavin et al., 2015).

Besides the cell source, various strategies, including both biological (transcription factor, growth factor and microenvironment) and biomechanical stimuli, have been applied to initiate/promote the *in vitro* tenogenic differentiation. For example, TGF beta signaling has been reported to be essential for tendon development (Havis et al., 2016; Pryce et al., 2009) and to promote the tendon differentiation of equine embryo-derived stem cells (Barsby and Guest, 2013). Animals with depletion of transcription factor SCX (Killian and Thomopoulos, 2016; Murchison et al., 2007; Yoshimoto et al., 2017), MKX (Ito et al., 2010; Liu et al., 2010; Onizuka et al., 2014; Suzuki et al., 2016), or Egr1 (Guerquin et al., 2013) showed aberrant and dysfunctional tendon. Ectopic expression of SCX converted human bone marrow derived mesenchymal stem cells (BMSCs) into tendon progenitor cells(Alberton et al., 2012), and in a rat patellar window injury model, the *Scx-*transduced TPSCs promoted tendon repair(Tan et al., 2014). Moreover, MKX over-expressed mesenchymal stem cells showed enhanced tenogenic differentiation potential through activation of the TGF beta signalling (Liu et al., 2015). It has also been reported that the cumulative mechanical loading was associated with mesenchymal stem cell-to-tenocyte differentiation(Morita et al., 2013; Morita et al., 2018) and cyclic tensile strain was able to induce the tenogenic differentiation of rat TPSCs (Xu et al., 2015). Furthermore, combination of biochemical and biomechanical cues synergistically enhanced the tenocyte/ligament cell differentiation of various types of cells (Chen et al., 2014; Chen et al., 2012; Nichols et al., 2018a; Nichols et al., 2018b).

During reprogramming process, fully differentiated somatic cells undergo significant epigenome rearrangements to re-establish the pluripotent stem cell network (Shipony et al., 2014). However, these rearrangements are insufficient to fully erase the parental epigenetic signatures, and iPSCs still retain a memory of their origin to some extent (Bar-Nur et al., 2011; Kim et al., 2010), which appears to have strong impact on their regeneration capacity (Hiler et al., 2015). In the present study, we aimed to generate tenocyte-derived iPSCs and to compare their tenogenic differentiation capacity with BMSCs by induction with mechanical loading and ectopic expression of transcription factor Mohawk. Our work should benefit both basic and clinic research on tendon repair with iPSCs.

## 2. Materials and methods

### 2.1. Isolation and culture of primary tenocytes, chondrocytes and BMSCs

Equine patellar tendon tissue or articular cartilage were surgically dissected from a two-year old horse. After thorough wash with phosphate buffered solution (PBS) containing 2× antibiotic/antimycotic solution (Gibco), the tissue was minced in basic medium (DMEM/F12 (Invitrogen) supplemented with 10% FCS (Gemini) and 1× antibiotic/ antimycotic solution), and then incubated with 2 mg/mL of collagenase D (Roche) in basic medium at 37 °C with gentle agitation. After 16 h, the digested materials were passed through a 100 μm strainer. The flow-through was then centrifuged at 200*g* for 10 min, and cell pellet was washed twice with medium. The cells were finally resuspended and cultured in basic medium at 37 °C under 5% CO_2_, and medium was changed every 2–3 days. At the confluency of 80–90%, cells were dissociated with 0.25% trypsin-EDTA, and sub-cultured at a density of 1–2 × 10^5^ cells/cm^2^.

For equine BMSCs isolation, bone marrow aspirates from three horses were collected individually in ACD solution (anticoagulant citrate dextrose solution) and washed twice with PBS followed by two more washes with basic medium at 170 g for 7 min each, and then resuspended and cultured in BMSC growth medium (basic medium plus 4 ng/mL bFGF) at 37 °C, 5% CO_2_. After 72 h, cells were thoroughly washed with PBS, and fresh medium was added with a change of every 2–3 days. Upon reaching 80–90% confluency, cells were dissociated with 0.25% trypsin-EDTA, and further expanded at a density of 1–2 × 10^5^ cells/cm^2^. BMSCs at passages 2–5 were used for experiments. Characterization of mesenchymal stem cell was carried out by flow cytometry with positive expression of CD29 (EMD Millipore, Cat# CBL481), CD44 (ThermoFisher Scientific, Cat#MA1–10229), CD90 (WSU Monoclonal Antibody Center, Item#DG2015), CD105 (Bio-Rad Laboratories, Cat#MCA1557A), MHC-I (gift from Dr. Douglas F. Antczak, Cornell University) and with negative expression of CD45 (WSU Monoclonal Antibody Center, Item#HR-DG2009), CD79 (Bio-Rad Laboratories, Cat#MCA2538A) and MHC-II (gift from Dr. Douglas F. Antczak).

### 2.2. Generation of iPSCs from equine tenocytes

Tenocyte-derived iPSCs were generated by using a single lentiviral stem cell cassette as previously described (Sommer et al., 2009). Briefly, pHAGE-STEMCCA lentiviruses expressing mouse Oct3/4, Sox2, Klf4, and c-Myc were produced in 293 T packaging cells, and supernatant containing the viral particles were filtered through 0.45 μm filter. Tenocytes were seeded on 35-mm culture plates at a density of 20,000 cells/cm^2^ the day before infection, and then incubated with viral particles for eight hours in the presence of polybrene B (8 μg/mL). Infected cells were maintained in basic medium for 30 h, and then transferred to mitomycin C inactivated MEF feeder cells in iPSC medium (DMEM containing 10% FCS, 1× NEAA, 1× L-glutamine, 1× sodium pyruvate, 0.055 mM beta-Mercaptoethanol, 1000 U/mL of LIF, and 1× antibiotic/antimycotic solution). Medium was replaced every other day. About 10–15 days individual colonies (designated as P0) were manually picked, trypsinized, and further expanded in 6-well plates pre-seeded with feeders in iPSC medium containing 10 μM of Rock inhibitor Y27632. Medium was replaced daily. When the cells reached about 80% confluence, they were mechanically dissociated, and passaged further or frozen in liquid nitrogen. At passages 3–5, cells were switched to and maintained in feeder-free StemFlex™ medium (Fisher Scientific). Cells at passages 10–25 were used in this study.

### 2.3. Karyotyping of teno-iPSCs

To harness metaphase cells, teno-iPSCs at 80–90% confluence were treated with 50 ng/mL nocodazole for fourhours, then collected at 150 g for 5 min after trypsin-EDTA treatment. Cells were resuspended in 3.7 mM KCl solution and incubated at 37 °C for 20 min. After two washes with fixative solution (glacial acetic acid/methanol in a ratio of 1:3), cells were spread onto microscope slides (Fisher Scientific). Chromosomes were visualized using DAPI staining under fluorescent microscope.

### 2.4. Immunofluorescent imaging

Fluorescence microscopy was performed as described in a previous study(Yang et al., 2012) with a few modifications. Briefly, cells seeded on glass coverslips were washed with PBS and fixed with 4% paraformaldehyde (PFA) in PBS for 20 min at room temperature. After permeabilization by incubation with 0.5% Triton X-100 in PBS for 5 min, cells were incubated with 2% bovine serum albumin (BSA) in PBS for one hour, followed by incubating overnight at 4 °C with primary antibodies: Oct3/4 (Santa Cruz Biotechnology Inc., Cat#sc-365,509), Sox2 (Cat#sc-17,320), Nanog (Cat#sc-134,218), TRA-1–81 (Invitrogen, Cat#MA1–024-D488), or Mohawk (Abcam, Cat#ab179597). Cells were stained with Alex Fluor 555-conjugated or Alex Fluor 488-conjugated 2nd antibodies (Abcam) for one hour at room temperature. Cellular DNA was stained with 4′,6-diamidino-2-phenylindole (DAPI, Molecular Probe, Eugene, OR). Fluorescence signals were detected on a Nikon Eclipse TE300 fluorescent microscope or on a Leica TCS SP5 confocal microscope.

### 2.5. Multi-lineage differentiation

For embryoid body formation, cells were dissociated with 0.5 mM EDTA in PBS, washed with basic media, then passed through 100 μM strainer and cultured in suspension for up to four weeks in ultra-low attachment 6-well plate (Fisher Scientific). Medium was changed every 2–3 days. Embryoid bodies were imaged on a Nikon eclipse TE300 and collected for gene expression analysis at desired time points.

For osteogenic differentiation, EDTA-dissociated cells were washed with basic medium and then seeded at a density of 1.2 × 10^4^ cells/cm^2^ in 12-well tissue culture plates. After three days, the medium was switched to either basic medium (as control) or differentiation medium, which contains basic media, 100 nM dexamethasone, 10 mM β-glycerophosphate, 50 μM ascorbic acid-2 phosphate. Cells were maintained in the differentiation medium for three weeks with medium change every 2–3 days. At the end of differentiation, samples were either collected for osteogenic gene expression analysis, or subject to Alizarin Red S (Sigma) staining and imaged on a Nikon eclipse TE300.

For chondrogenic differentiation, EDTA-dissociated cells were washed with basic medium and then seeded at a density of 1.2 × 10^4^ cells/cm^2^ in 12-well tissue culture plates. After three days, the medium was changed to chondrogenic medium, which was composed of basic medium, 50 μg/mL ascorbic acid, 100 nM dexamethasone, 1× ITS premix (BD Biosciences), 40 μg/mL L-proline, and 10 ng/mL human recombinant TGF-β3 (R&D systems). Cells were fed with fresh differentiation medium every 2–3 days. At 21 days, cells were collected for chondrogenic gene expression analysis, or subject to Alcian blue staining and imaged on a Nikon eclipse TE300.

For adipogenic differentiation, EDTA-dissociated cells were washed with basic medium and then seeded at a density of 1.2 × 10^4^ cells/cm^2^ in 12-well tissue culture plates. After three days, cells were cultured in basic medium containing 1 μM dexamethasone, 0.5 mM 3-IBMX, 0.1 mM indomethacine and 1.7 μM insulin for 1 week with medium change every 2–3 days, then maintained in medium consisting of basic medium and 1.7 μM insulin for another two weeks. At the end of differentiation, cells were harvested for adipogenic gene expression analysis or subject to Oil-red O staining (Sigma-Aldrich).

### 2.6. Mechanical loading[42]

To test the effects of mechanic force on tenogenic differentiation of BMSCs and teno-iPSCs, a customized bioreactor was used to apply cyclic uniaxial sinusoidal deformations to cell-seeded poly(ɛ-caprolactone) (80 kDa; Sigma-Aldrich, St. Louis, MO) nanofibrous scaffolds during *in vitro* culture. The device was programmed to approximate sinusoidal waveforms equating to 3% strain amplitude (0%–6% strain) at a frequency of 1.0 Hz for 18 h. At the end of mechanical stretching, samples were either collected for RNA extraction, or fixed in 4% PFA for immunofluorescent staining.

### 2.7. Gene expression analysis by reverse transcription PCR (RT-PCR) or quantitative real-time PCR (qPCR)

Samples were lysed in trizol (Invitrogen), and total RNA was extracted according to the manufacturer’s instruction. One microgram of RNA was treated with RQ1 RNase-free DNase (Roche), and then used for complimentary DNA (cDNA) synthesis by using High-Capacity cDNA Reverse Transcription Kit (Thermo Fisher Scientific). Equine specific primer pairs were designed using NCBI primer-blast or published data (Bavin et al., 2015), and the list of primer sequences can be found in Supplemental Table 1. Standard RT-PCR was conducted with 10 ng of cDNA per reaction by using Hot Start Taq 2× Master Mix (New England Biolabs, USA) on an Eppendorf Mastercycler Nexus Thermal Cycler (Fisher Scientific, USA), and qPCR was carried out with 5 ng of cDNA per reaction using SYBR Green PCR Master Mix (Fisher Scientific, USA) on an Applied Biosystems 7500 real time PCR system. All PCR reactions were performed in duplicates, and qPCR cycling was started with 95 °C for 10 min, followed by 40 cycles of 95 °C for 15s, 60 °C for 15 s, and 72 °C for 15s. At the end of the program, a melt curve was produced by taking readings every 1 °C from 65 °C to 95 °C. The reference gene GAPDH was used to normalize gene expression levels between groups by 2^−ΔCt^ method.

### 2.8. Construction of equine Mohawk-expressing vectors

To clone equine *Mkx* gene, primers were designed based on online predicted mRNA sequence for *Equus caballus* mohawk homeobox (accession number: XM_014737017). Total mRNA was extracted from tendon tissue, and cDNA was synthesized as mentioned above. PCR was performed using Platinum PCR SuperMix High Fidelity (Invitrogen) with primer pairs (Forward: 5′- ACGA AGATCTATGCGGGAA GTGGGT CGGCGCGGGGCTG-3′; Reverse:5’-GAGCGTCGACGGG TTTCAGTCCTG GAATGGTTCG-3′). The PCR products were then cloned into *Bgl*II and *Sal*I sites of eGFP-C1 vector, and further sub-cloned into pHAGE lentiviral vector. Individual clones with *Mkx* gene were confirmed by DNA sequencing. Of note, the accession number for predicted *Mkx* mRNA sequence used here is different from current updated version XM_023632370 or XM_023632373.

### 2.9. Mohawk short hairpin RNA (shRNA) interference

The shRNA oligonucleotides contained sense strands of equine *Mkx* nucleotide sequences, followed by short spacers (TTCAAGAGA), the reverse complement of the sense strands, and six thymidines as RNA polymerase III transcriptional stop signal. The detailed oligonucleotide sequences, targeting the open reading frame (shMKX) and 3′-untranslated region (shM3U) of equine *Mkx* gene, are shown in Supplemental table 2. The oligonucleotides were annealed and ligated into the *Hpa*I and *Xho*I sites of pLB vector (Addgene plasmid#11619). All the plasmids were confirmed by sequence analysis. Replication-defective lentiviruses were produced in 293 T packaging cells, and supernatant containing viral particles were harvested at 48, 60, and 72 h and filtered through a 0.45 μm PVDF filter (Millipore, Ireland). Cells were exposed to viral supernatant at 1:1 ratio for six hours in the presence of Polybrene B (8 μg/mL). The infection efficiency was evaluated by the percentage of green fluorescent protein (GFP)-positive cells under a fluorescent microscope.

### 2.10. Western blot

Cells were lysed in RIPA buffer on ice, and protein concentration was quantitated using Bio-Rad Protein Assay (Bio-Rad Laboratories, USA, Cat#5000006). SDS-PAGE was carried out using a mini gel system from Bio-Rad. Proteins were transferred to PVDF membranes. After blocking with TBST (Cell Signaling Technology, USA) containing 5% nonfat dry milk (blocking buffer) for at least one hour at room temperature, the membranes were incubated at 4 °C overnight with primary antibodies at a dilution of 1:1000 in the blocking buffer, followed by incubation with horseradish peroxidase-conjugated secondary antibodies (Abcam, USA) for one hour at room temperature. After thorough wash with TBST buffer, signals on the membranes were developed with an enhanced chemiluminescent system (Pierce, USA).

### 2.11. Statistics

Data were expressed as means ± SD. Anova single factor test was used to determine the statistically significant differences in gene expression among different cell types. Paired student’s *t*-test was used to determine statistically significant differences in gene expression between the control and treated groups. Significance was shown as * (*P* < .05) or ** (*P* < .01).

## 3. Results

### 3.1. Generation and characterization of tenocyte-derived iPSCs

**Fig. 1.**
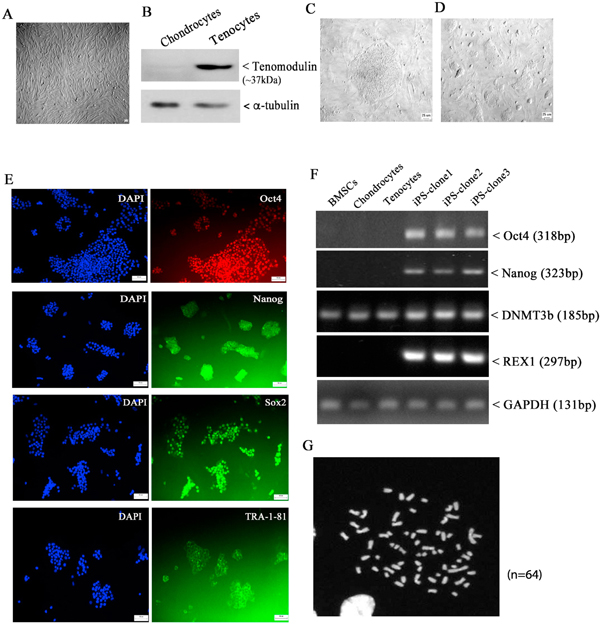
Generation of iPSCs from adult tenocytes. A: morphology of primary tenocytes (at passage 3) isolated from equine patellar tendon tissue (scale bar = 10μm); B: chondrocytes and tenocytes were lysed in RIPA buffer, and equal amounts of cell lysates were immunoblotted with antibodies against α-tubulin and tenocyte marker Tenomodulin. C: Formation of teno-iPSC colony after infection of tenocytes with a single lentiviral stem cell cassette (scale bar = 25μm); D: expansion of teno-iPSC on mouse embryonic fibroblast feeder cells (scale bar = 25μm); E: teno-iPSCs cultured in 12-well plate were fixed with PFA for immunofluorescent staining with antibodies against stem cell markers Oct4, Nanog, Sox2, or Tra-1–81 (scale bar = 25 μm). F: RT-PCR showing the expression of stem cell markers *POU5F1*, *NANOG*, *DNMT3B* and *REX1* in teno-iPSCs; G: teno-iPSCs (passage 5) maintains normal equine karyotype (*n* = 64).

It has been reported that the origin of somatic cell type affects the differentiation potential of the resulting iPSCs(Polo et al., 2010). Tenocytes are the major cells found throughout the tendon structure and essential for the production of extracellular tendon matrix proteins. Cells isolated from adult equine patellar tendon expressed abundant tenocyte marker tenomodulin, which was barely shown in equine chondrocytes (Fig. 1A&B). After infection of tenocytes with a single lentiviral stem cell cassette system, visible colonies appeared at day 7, and the colonies with appropriate size were individually picked and expanded (Fig. 1C&D). Immunofluorescent staining with antibodies against Oct4, Sox2, Nanog, and TRA-1–81 confirmed the expression of pluripotency markers in the resulting iPSCs (Fig. 1E & S1). Moreover, RT-PCR with equine-specific primer pairs showed high levels of *Oct4 Nanog*, and *REX1* in all the tested iPSC clones, which were not detected in the primary tenocyte cultures (Fig. 1F). Compared to donor tenocytes, iPSCs showed increased mRNA level of *DNMT3b* (Fig. 1F). Additionally, normal karyotype (*n* = 64, Fig. 1G) indicated the maintenance of genomic stability in the iPSCs.

**Fig. 2.**
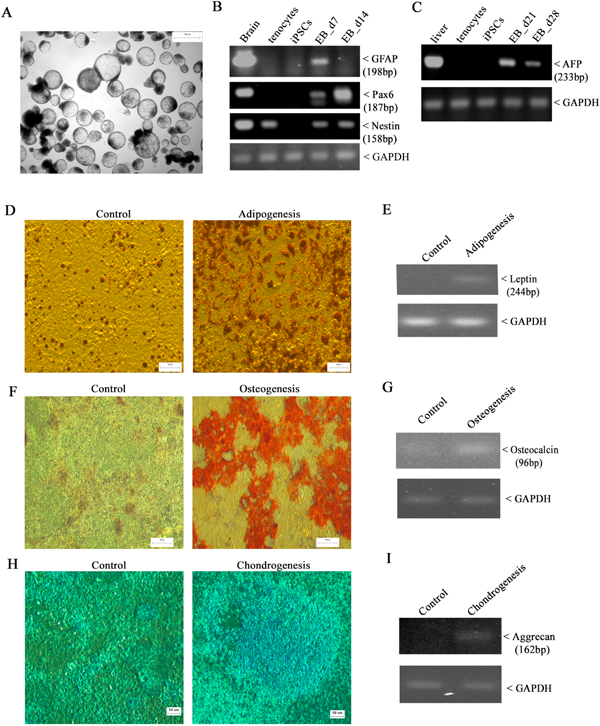
Multi-lineage differentiation capacity of teno-iPSC. A: morphology of 7-day old embryoid bodies (EBs) formed in suspension culture (scale bar = 100 μm); B: RT-PCR analysis showing the expression of ectodermal markers Glial fibrillary acidic protein (*GFAP*), *Pax6*, and Nestin (*NES*) in teno-iPSC-derived EBs at days 7 and 14; C: RT-PCR analysis showing the expression of endodermal marker α-fetoprotein (*AFP*) in teno-iPSC-derived EBs at days 21 and 28; D&E: *in vitro* adipogenic differentiation of teno-iPSCs. The fat droplets were displayed by oil-red staining (scale bar = 200 μm), and the induction of adipocyte marker leptin (*LEP*) was assessed by RT-PCR; F&G: *in vitro* osteogenic differentiation of teno-iPSCs. The calcium deposition was revealed by Alizarin Red S staining (scale bar = 200μm), and the expression of osteocalcin (*BGLAP*) was measured by RT-PCR; H&I: *in vitro* chondrogenic differentiation of teno-iPSCs. The production of proteoglycan proteins was shown by Alcian blue staining (scale bar = 50μm), and the induction of aggrecan (*ACAN*) was determined by RT-PCR. (For interpretation of the references to colour in this figure legend, the reader is referred to the web version of this article.)

To determine their pluripotency, iPSCs were cultured in suspension to spontaneously form embryoid bodies (EBs, Fig. 2A), and the expression of germ layer-specific genes was measured by RT-PCR (Sheridan et al., 2012). As expected, ectodermal markers *GFAP* and *Pax6* were activated in EBs at days of 7 and 14, which were barely detected in either donor tenocytes or the resulting iPSCs (Fig. 2B). Notably, Nestin (*NES*), another ectodermal marker, was expressed not only in EBs, but also in tenocytes. This is not surprising as a sub-population of nestin^+^ cells has been identified within the tendon cell population(Yin et al., 2016). On the other hand, the expression of endodermal marker alpha-fetoprotein (*AFP*) was evident in EBs at days of 21 and 28, which was negligible in tenocytes and iPSCs (Fig. 2C). When iPSCs were cultured under defined condition for adipogenesis, oil-red staining showed the formation of lipid droplet, and the expression of adipogenesis marker Leptin was detected at mRNA level (Fig. 2D&E). Culture of iPSCs with osteogenesis medium for three weeks resulted in significant calcium deposit, along with increased expression of osteocalcin (*BGLAP,* Fig. 2F&G). When cells were cultured in chondrogenesis medium, the production of proteoglycan proteins was visualized by positive Alcian blue staining, and the expression of Aggrecan (*ACAN*) was detected by RT-PCR (Fig. 2H&I). Taken together, these data confirmed that our tenocyte-derived iPSCs (teno-iPSCs) have the capacity to differentiate into other lineages.

### 3.2. Altered tenocyte-lineage gene expression in teno-iPSCs

**Fig. 3.**
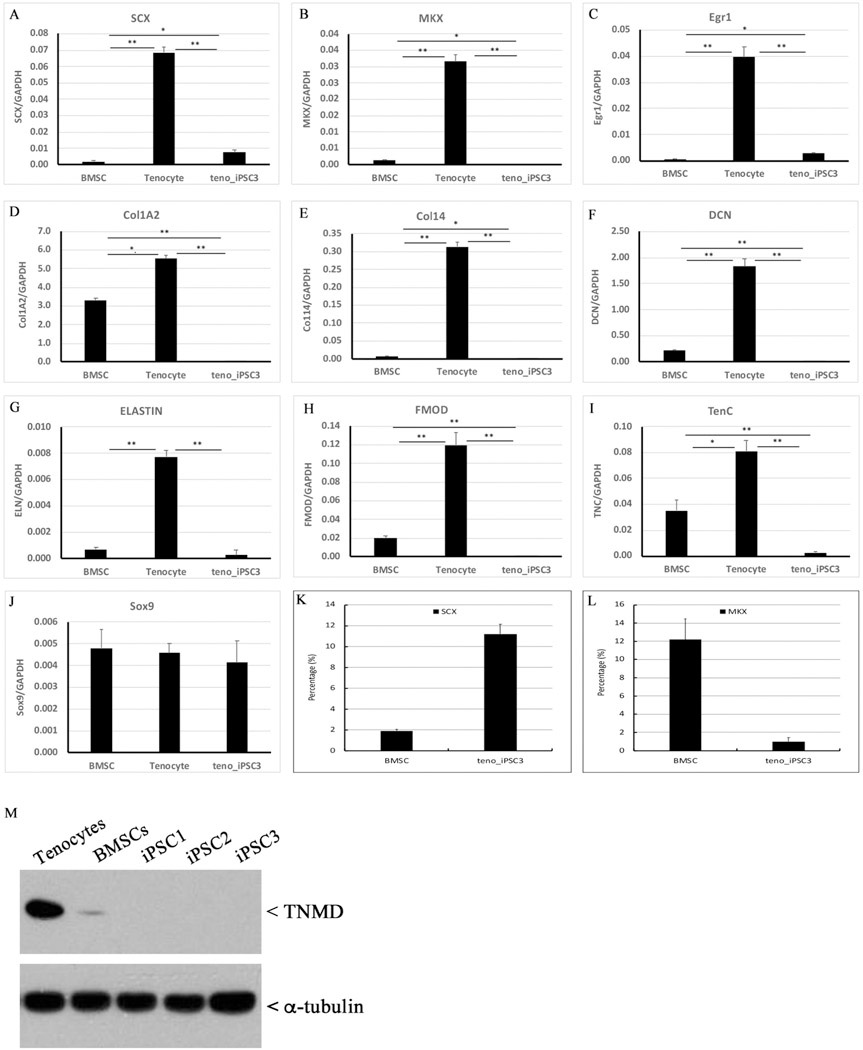
Alteration of tenocyte-linked gene expression in teno-iPSCs. A-J: cDNA samples were prepared from BMSCs, tenocytes, and teno-iPSCs, and the expression of tenocyte-linked genes, including *Scx*, *Mkx*, *Egr1*, *Col1A2*, *Col14*, *DCN*, *ELN*, *FMOD*, *TNC,* and *Sox9*, was determined by qPCR; K&L: relative transcriptional activity of *Scx* and *Mkx* in BMSCs and teno-iPSCs (normalized to tenocytes); M: Cells were lysed in RIPA buffer, and equal amounts of cell lysates were blotted with antibodies against α-tubulin and TNMD.

To determine whether our teno-iPSCs retained active expression of lineage-specific genes from donor cells, a panel of tenocyte-linked genes in teno-iPSCs and parental tenocytes were measured by qPCR. As expected, *Scx*, *Mkx*, *Egr1*, *Col1A2*, *Col14*, *DCN*, *ELN*, *FMOD,* and *TNC* were highly expressed in donor tenocytes (Fig. 3A–I). However, those genes were significantly suppressed in teno-iPSCs (Fig. 3A–I). Interestingly, the mRNA levels of the above genes varied among individual iPSC clones (Fig. S2), despite the fact they were derived from the same donor horse. Since the Clone 3 showed overall higher levels of tenocyte-linked genes among the three clones, it was chosen for further study. As a comparison, we also measured the tenogenic gene expression in equine BMSCs in parallel with teno-iPSCs through the study. As shown in Fig. 3D–I, except *ELN*, all other tested tendon-related extracellular matrix (ECM) genes, including *Col1A2*, *Col14*, *DCN*, *FMOD*, and *TNC,* were expressed at much lower level in teno-iPSCs than those in BMSCs. As to the tenogenesis-regulating transcription factors, the mRNA level of *Scx* in teno-iPSCs was about 1/10 of that in tenocytes and about 3 times of that in BMSCs (Fig. 3K–L), whereas the mRNA level of *Mkx* in teno-iPSCs was only about 1/100 of that in tenocytes and about 1/12 of that in BMSCs (Fig. 3K–L). For unknown reason, RT-PCR with three sets of primer pairs failed to detect *TNMD* expression in any type of cells used in this study. However, immunoblotting with antibodies against tenomodulin (TNMD) protein showed specific signal at expected size for cell lysates from tenocytes and BMSCs, and this signal was absent in all three tested teno-iPSC lines (Fig. 3M). Taken together, these data strongly indicate that the reprogramming process initiated by Yamanaka factors have significantly altered the tenogenic gene transcriptional activities in the resulting teno-iPSCs.

### 3.3. Regulation of tenogenic gene expression by mechanical loading

**Fig. 4.**
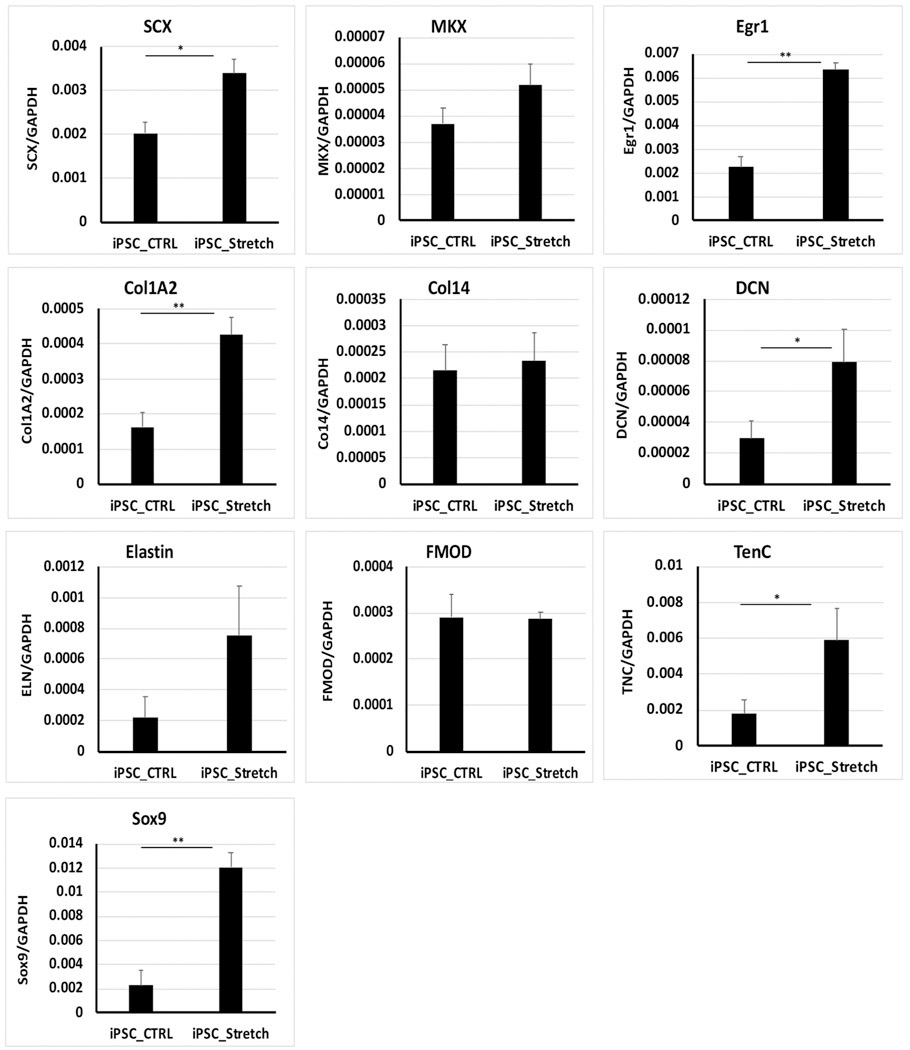
Expression of tenogenic genes in mechanical force loaded teno-iPSCs. Teno-iPSCs were seeded on vitronectin-coated PCL scaffolds for two days in basic medium, and then subject to uniaxial mechanical stretching for 18h (iPSC_Str). Cells seeded on PCL scaffolds without mechanical loading were served as control (iPSC_CTRL). Gene expression was determined by qPCR;

So far, there is still no well-defined condition for the tenogenic differentiation of either MSCs or ESCs. It has been recognized that mechanical force plays important roles in tendon development and that *in vitro* mechanical loading can potentially induce tenogenic gene expression in mesenchymal stem cells(Filomeno et al., 2012; Galloway et al., 2013; Liu et al., 2017; Song et al., 2010). Consistent with this notion, when equine BMSCs were subject to uniaxial mechanical force for 18 h, the expression of *Scx*, *DCN*, *TNC,* and the mechanosensitive transcription factor *Egr1* was significantly increased, and the expression of *Mkx*, *Col1A2*, *Col14,* and *ELN* also trended upwards (Fig. S3). When teno-iPSCs underwent mechanical stretching, the expression of *Scx*, *Egr1*, *Col1A2*, *DCN,* and *TNC* was notably elevated, while the increases for *Mkx* and *ELN* were not significant (Fig. 4). Taken together, these data suggest that, like BMSCs, the tenogenic gene activities in teno-iPSCs can be regulated by mechanical loading.

### 3.4. Mohawk regulates tenogenic gene expression in tenocytes and BMSCs

Transcription factor MKX has been reported to play critical roles in tendon development through regulating the expression of tendon extracellular matrix genes(Huang et al., 2015). In our study, knockdown of *Mkx* by shRNA targeting either open-reading frame (shMKX) or 3′-untranslated region (shM3U) of *Mkx* gene indeed decreased mRNA levels of *Scx*, *Mkx*, *Egr1*, *Co1A2*, *Col14*, *DCN,* and *FMOD* in either equine tenocytes or BMSCs (Fig. S4 & S5). Interestingly, knockdown of *Mkx* downregulated the expression of *ELN* in tenocyts but not in BMSCs. Moreover, in both tenocytes and BMSCs, knockdown of *Mkx* upregulated the expression of *Sox9*, a known chondrogenesis master regulator. Efficient knockdown of *Mkx* was also confirmed by immunoblotting of cell lysates with antibodies against MKX (Fig. S4 &S5). These data support the notion that MKX is required for the regulation of tenogenic gene expression in BMSCs and tenocytes.

### 3.5. Cloning of equine Mkx gene

**Fig. 5.**
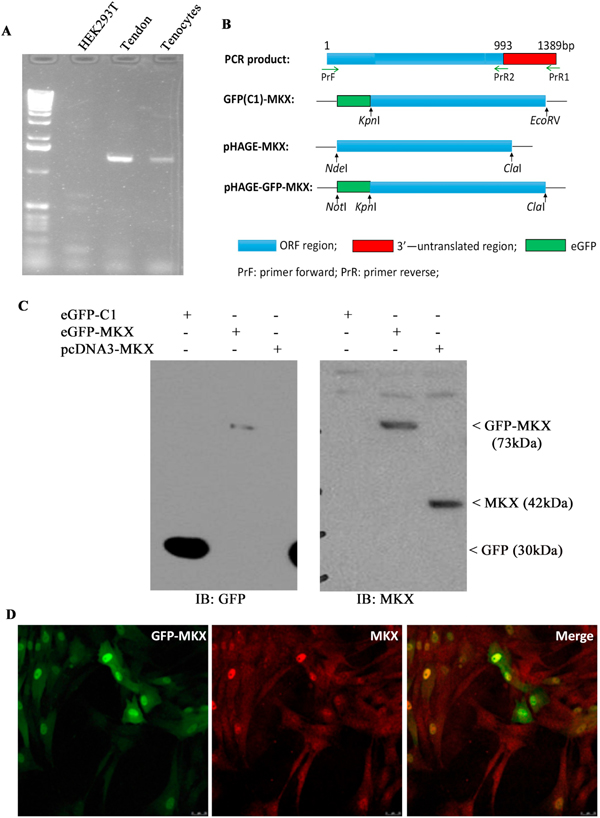
Cloning of equine *Mkx* gene. A) Detection of Mohawk expression by PCR in HEK293T cells, patellar tendon tissue and adult tenocytes; B) diagram for construction of MKX-expressing plasmids; C) HEK293T cells were transiently transfected with plasmids expressing GFP alone, or GFP-tagged MKX, or MKX alone for 48 h, and then lysed in RIPA buffer. Equal amounts of cell lysates was blotted for GFP and MKX; D) BMSCs were infected with pHAGE-GFP-MKX lentivirus for two days, then seeded on glass coverslips for another two days before being fixed for immunofluorescent staining with antibodies against MKX. Signals were detected on a Leica TCS SP5 confocal microscope. Scale bar =25 μm.

As mentioned above, the expression of *Mkx* and most of the tendon-related ECM genes was highly suppressed in teno-iPSCs (Fig. 3). Although the response to mechanical loading was evident, the relative mRNA levels of the tested tenogenic genes in stimulated teno-iPSCs were still lower than those in BMSCs (Fig. 4&S3). We then asked whether overexpression of Mohawk into teno-iPSCs would boost the expression of ECM genes and facilitate their tenogenic differentiation. To this purpose, total mRNA isolated from tendon tissue was used to clone equine Mohawk gene. The PCR products were sub-cloned into mammalian expression eGFP-C1 vector or pHAGE-lentiviral vector (Fig. 5A–B). After transfection of HEK293T cells with plasmids expressing MKX tagged with or without GFP, immunoblotting of whole cell lysates with antibodies against MKX and GFP-tag confirmed the ectopic expression of MKX as well as the specificity of Mohawk antibody (Fig. 5C). Moreover, as expected, immunofluorescent staining of BMSCs infected with lentivirus expressing GFP-tagged MKX displayed apparent nuclear localization of GFP signals that were co-localized with MKX signals (Fig. 5D). Taken together, these data confirmed the success in cloning of equine *Mkx* gene.

### 3.6. Regulation of tenogenic gene expression by re-introduction of Mohawk

**Fig. 6.**
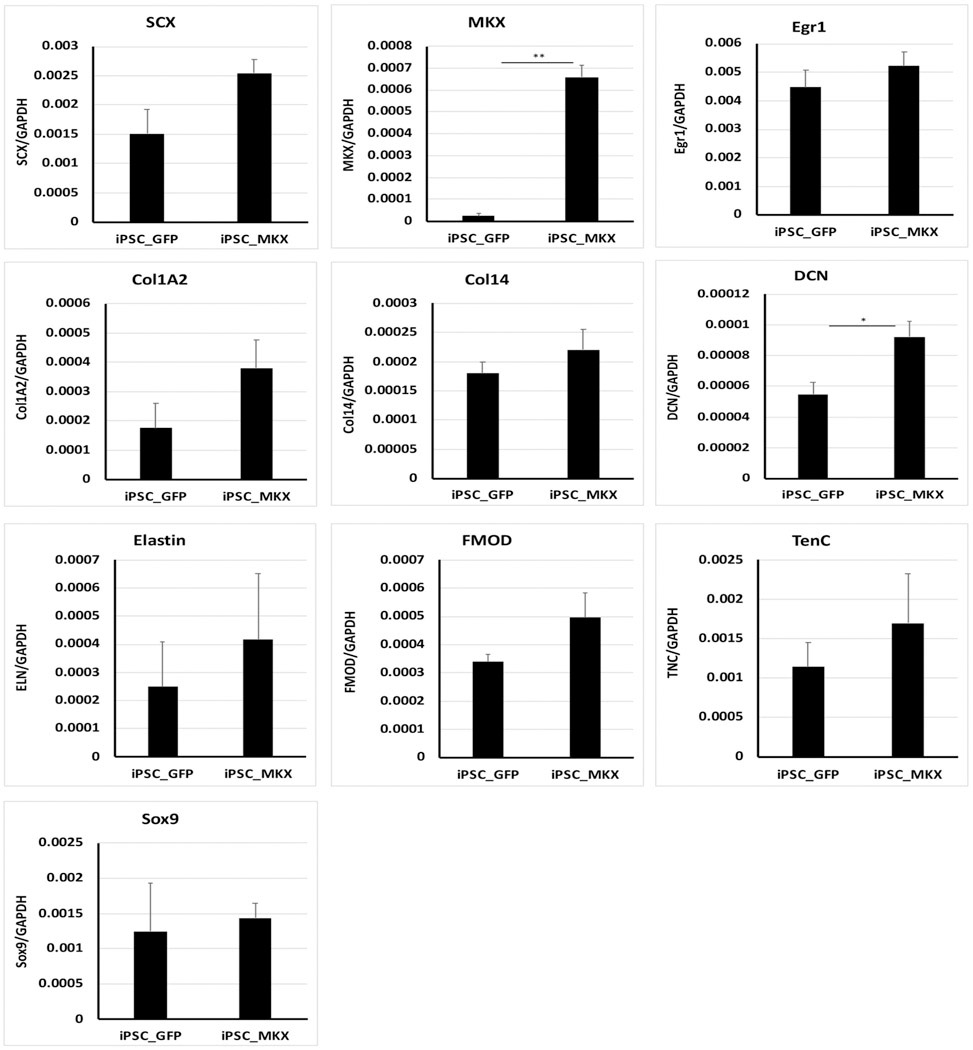
Expression of tenogenic genes in *Mkx*-transduced teno-iPSCs. Teno-iPSCs were infected with pHAGE-MKX (iPSC-MKX) or control pHAGE-GFP (iPSC-GFP) for seven days in basic medium, and the expression of tenocyte-linked genes was measured by qPCR.

To study the effects of MKX on tenogenesis, BMSCs were infected with MKX-expressing lentivirus for seven days, and the expression of tenogenic genes was measured. qPCR revealed a significant increase of *Scx*, *Mkx*, *Col14,* and *FMOD* (Fig. S6). Furthermore, exposure of MKX-ectopically expressed BMSCs to lenti-shMKX downregulated the expression of *Scx*, *Egr1*, *Col1A2*, *Col14*, *DCN*, *ELN*, *FMOD,* and *TNC*, which was not observed by infection with lenti-shM3U (Fig. S7). The ectopic expression of MKX and efficient knockdown of endogenous MKX by shM3U were confirmed by immunoblotting with antibodies against MKX (Fig. S7). These data not only demonstrate the biological function of the cloned equine *Mkx* gene, also indicate the essential role of Mohawk in regulating tenogenic gene expression in BMSCs. To determine the role of MKX on tenogenic gene expression in teno-iPSCs, cells were infected with MKX-expressing lentivirus for seven days, and the expression of tenogenesis-related genes were analyzed by qPCR. As expected, the expression of *Mkx* was strongly increased, and its level was comparable to that in the primary BMSCs (Fig. 6). Interestingly, ectopic expression of *Mkx* also resulted in significant elevation of *DCN* in teno-iPSCs. The expression of *Scx*, *Egr1*, *Col1A2*, *Col14*, *ELN*, *FMOD,* and *TNC* was increased, but not significant (Fig. 6).

### 3.7. Synergistic effects of Mohawk and mechanical loading on tenogenic gene expression

**Fig. 7.**
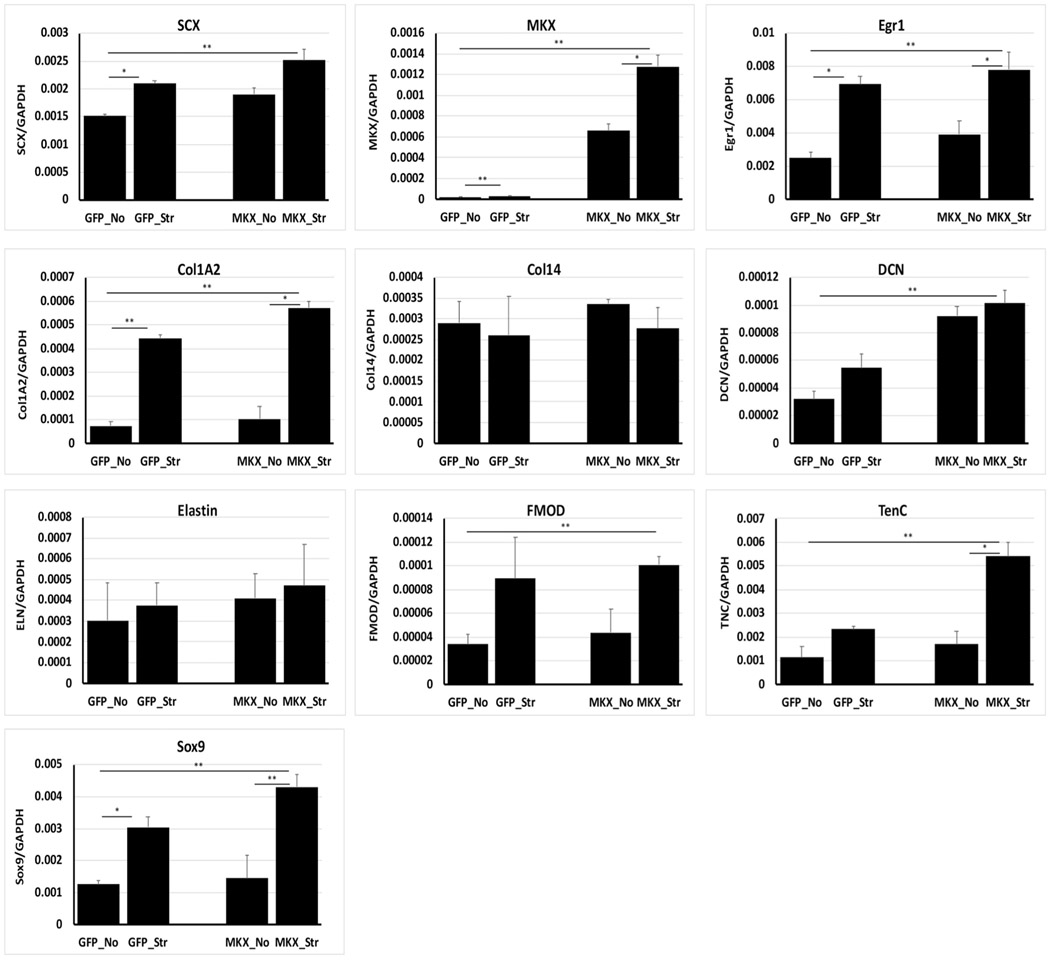
Synergistic effects of Mohawk and mechanical stretching on tenogenic gene expression in teno-iPSCs. Teno-iPSCs were infected with pHAGE-MKX or control pHAGE-GFP for four days, then split and seeded on vitronectin-coated PCL scaffolds for another two days before being subject to mechanical stretching for 18 h (GFP_Str; MKX_Str). Cells seeded on PCL scaffolds without mechanical loading were served as control (GFP_No; MKX-No). Expression of tenocyte-linked genes was determined by qPCR.

To test whether the tenogenic gene expression can be synergistically regulated by MKX and mechanical loading, MKX-expressing BMSCs and teno-iPSCs were subject to mechanical loading for 18 h, and the expression of tenogenic genes was assessed by qPCR. As expected, the mRNA levels of *Egr1* in both BMSCs and teno-iPSCs were augmented upon mechanical loading. Ectopic expression of *Mkx* further increased the mechanical stimulation-induced expression of *Scx*, *Egr1*, *Col1A2*, *DCN,* and *TNC* in teno-iPSCs (Fig. 7) and that of *Scx*, *Egr1*, *DCN,* and *TNC* in BMSCs (Fig. S8). These data suggest that the combination of *Mkx* overexpression and mechanical stretching may have synergistic effects on facilitating the tenogenic gene expression in teno-iPSCs and BMSCs.

## 4. Discussion

In this study, by using a single lentiviral stem cell cassette expressing four Yamanaka transcription factors, we generated equine tenocyte-derived iPSCs which showed multi-lineage differentiation capacity. Compared to parental tenocytes, the resulting teno-iPSCs expressed significantly lower levels of lineage-specific genes, including *Scx*, *Mkx*, *Egr1*, *Col1A2*, *Col14*, *DCN*, *ELN*, *FMOD,* and *TNC*. These repressed genes, except *Col14* and *ELN,* could be re-activated by cyclic uniaxial mechanical loading and ectopically transduced transcription factor *Mkx* alone or combined.

### 4.1. Altered lineage-specific transcriptional activity in teno-iPSCs

Although it is believed that successful reprogramming of differentiated somatic cells requires complete erasure of the parental epigenetic signature(Nashun et al., 2015), retention of epigenetic memory from the donor cells has also been reported(Bar-Nur et al., 2011; Kim et al., 2010; Phetfong et al., 2016; Roberts et al., 2017). While this might be a problem for the differentiation of iPSCs into lineages different from the donor cells, it might be advantageous for re-differentiation of iPSCs into the same lineage. Indeed, human osteoblast cells derived iPSCs were able to differentiate into osteoblast progenitors (Roberts et al., 2017). In our study, reprogramming of tenocytes with traditional Yamanaka factors significantly repressed the transcriptional activity of all the tested tenocyte-lineage specific genes, indicating the re-establishment of transcriptional network in the resulting iPSCs. Kyttaelae et al. reported that iPSC lines derived from the same donor were transcriptionally similar to each other(Kyttala et al., 2016), but our results demonstrated varied mRNA levels of parental lineage genes among the three tested teno-iPSC clones, implying that the retention degree of parental gene activities differed from one another. Kilpinen et al. suggested that many factors, such as cell type of origin, culture condition and reprogramming method, contributed to the variations between iPSC lines.(Kilpinen et al., 2017). One possible reason for the transcriptional variations among our teno-iPSC clones could be due to the heterogeneity nature of the primary tenocyte cultures, even though they were originally derived from the same donor horse. It is also noteworthy that, although teno-iPSCs displayed significant changes on the parental lineage-specific transcriptional network, they might still be inclined to re-differentiate to the same lineage than to others. Since Clone 3 showed higher level of tenogenic gene expression than other two clones, we assumed that this line might possess higher tenogenic differentiation potential. Further study will be necessary to understand whether and how variations among teno-iPSC clones affect their isogenic differentiation capacity.

### 4.2. Mechanical stretching alone on tenogenic differentiation

Tendon is a mechanosensitive tissue transferring force from muscle to bone. Biomechanical force plays critical roles in normal tendon development as well as in tendon repair(Galloway et al., 2013; Aspenberg, 2007; Brown et al., 2014; Snedeker and Foolen, 2017). Various types of cells and different mechanical loading parameters have been tested for mechanical force-induced tenogenic differentiation (Murchison et al., 2007; Song et al., 2010; Scott et al., 2011; Youngstrom et al., 2016; Zhang et al., 2010). Xu et al. reported that the cyclic tensile strain with different amplitudes (2%, 4%, and 8%) and frequencies (0.2 Hz, 0.5 Hz, and 1.0 Hz) had no influence on TPSC viability but showed different effects on the proliferation and the expression of tenogenic genes including *Col1*, *TNC*, *TNMD,* and *Scx(*Xu et al., 2015*)*. Nam et al. also reported that cyclic uniaxial loading with 4% strain and 1.0 Hz frequency enhanced human BMSC proliferation, but higher strains were required for the superior expression of tenogenic genes, including *COL1*, *COL3*, *TNC*, *Scx*, *DCN,* and *TNMD (*Nam et al., 2015*)*. In our study, the mechanical loading parameters (1.0 Hz with 0%–6% sinusoidal wave of strain) were also used in previous study (Baker et al., 2011). The cyclic uniaxial stretching upregulated the expression of *Egr1* in both BMSCs and teno-iPSCs, which was expected as EGR1 is a known biomechanical sensor(Gaut et al., 2016) and thus can be served as a marker for the effective mechanical loading on the targeting cells. Furthermore, the mechanical stretching also increased the expression of *Scx*, *DCN,* and *TNC* in BMSCs and teno-iPSCs. This agrees with the findings in other studies where human BMSCs were subject to mechanical strain for 7 or 14 days(Xu et al., 2015; Nam et al., 2015; Qiu et al., 2016). Interestingly, mechanical loading elevated collagen I gene expression in rat TPSCs (Xu et al., 2015) but not in human BMSCs (Qiu et al., 2016). Our data revealed an apparent increase of *Col1A2* expression in teno-iPSCs but not in BMSCs, indicating there might be different regulatory networks on *Col1A2* gene activity between these two types of cells. In addition, a significant increase of chondrogenic marker *Sox9* was detected in teno-iPSCs upon mechanical stretching (Fig. 4). Given to the low transcriptional activity of *Mkx* in teno-iPSCs, this result is in line with the report from another study where *Mkx*^*−/−*^ tendon-derived cells also showed increased expression of *Sox9* after mechanical stimulation.(Suzuki et al., 2016).

### 4.3. Transcriptional regulation on tenogenic differentiation

Mohawk is highly expressed in developing tendon and appears to be an important regulator of tenogenic differentiation, as *Mkx*^*−/−*^ mice and *Mkx*^*−/−*^ rats display marked tendon phenotype(Ito et al., 2010; Liu et al., 2010; Onizuka et al., 2014; Suzuki et al., 2016). However, it is unclear whether the phenotype is a direct cause from the loss of MKX-regulated ECM gene activity or an indirect cause from the change of ECM-regulating transcription factor(s) by the depletion of MKX. Although Liu et al. reported that, compared to wild littermates, there was no changes on the expression of *Scx* in *Mkx*^*−/−*^ neonatal tendon cells(Liu et al., 2010), another study with a *Mkx*^*−/−*^ mouse model showed significant increase of *Scx* in 8-week old Achille tendon than that in the wildtype(Pryce et al., 2009). Contradictory to the above report, the expression of *Scx* in the patellar tendon of 3-week old *Mkx*^*−/−*^ rat was significantly lower than that in the wildtype (Killian and Thomopoulos, 2016). In our study, knockdown of endogenous MKX using lentiviral-shRNA downregulated the expression of *Scx* in both BMSCs and tenocytes but not in *Mkx*-transduced BMSCs, strongly suggesting that MKX regulates the expression of of *Scx* at the transcription level. Furthermore, the expression of *Egr1*, which was not explored in *Mkx*^*−/−*^ animal models, was decreased in BMSCs and tenocytes after knockdown of MKX, indicating an interdependent transcriptional regulation network among tenogenesis-related transcription factors. Very likely, it was the loss of those key tenogenic transcription factors that caused the significant decrease of ECM gene expression, such as *Col1A2*, *Col14,* and *FMOD* in BMSCs, and *Col1A2*, *Col14*, *DCN*, *ELN,* and *FMOD* in tenocytes. Of note, reduction of *FMOD* expression was observed in the 3-week old *Mkx*^*−/−*^ rat patellar tendon (Suzuki et al., 2016) but not in 8-week old *Mkx*^*−/−*^ mouse Achille tendon (Ito et al., 2010). Additionally, compared to knockdown of *Mkx*, ectopic expression of *Mkx* seems to have less impact on the regulation of downstream ECM gene activities, except that, among the tested ECM genes, only *Col14* and *FMOD* in BMSCs, and *DCN* in teno-iPSCs, were highly up-regulated after *Mkx* transduction. In other words, ectopic expression of *Mkx* alone may be insufficient to induce tenogenic gene expression in BMSCs and teno-iPSCs.

### 4.4. Combination of biochemical and biomechanical cues in tenogenic regulation

It has been reported overexpression *Scx* combined with mechanical force have a synergistic effect on promoting the commitment of human ESC-derived MSCs to tenocytes (Chen et al., 2012). Another study by Nichols et al. also showed that combination of transient *Scx* overexpression with cyclic strain enhanced the differentiation of murine MSC line C3H10T1/2 into ligament-like cells (Nichols et al., 2018b). In a *Mkx*^*−/−*^ rat model, mechanical stretching enhanced the chondrogenic gene expression in tendon-derived cells(Ito et al., 2010; Suzuki et al., 2016), suggesting that MKX may play a role in regulating mechanical force-sensitive gene activities and thus affecting the cell fate. Indeed, our results demonstrated that the mechanical stimulation combined with ectopic expression of *Mkx* significantly increased the expression of *Scx*, *Egr1*, *Col1A2*, *DCN*, *FMOD,* and *TNC* in teno-iPSCs (Fig. 7) and that of *Scx*, *Egr1*, *Col1A2*, *Col14*, *DCN*, *FMOD,* and *TNC* in BMSCs (Fig. S7). On the other hand, whereas ectopic expression of *Mkx* did not influence the mechanical loading-induced expression of *Sox9* in teno-iPSCs (Fig. 6 &7), the levels of other chondrogenic-related genes *ACAN* and *Col2A1* were not upregulated by either ectopic expression of *Mkx* or mechanical loading (unpublished data). It is also worthy to mention that, as a marker for mature tenocytes, tenomodulin was not detected in cell lysates from either teno-iPSCs alone, or mechanical loading-stimulated teno-iPSCs, or *Mkx*-transduced teno-iPSCs. Although the underlying mechanism is unclear, the lack of tenomodulin expression could be a reflection of the persistent colony-like morphology of teno-iPSCs under the conditions used in this study (unpublished observation). Stimulation with soluble cytokines and cultivation in matrix-based environment may be necessary to overcome the challenge(Yang et al., 2017; Barsby et al., 2014). Nevertheless, our data indicate the tenogenic differentiation capacity of tenocyte-derived iPSCs. Future work will focus on investigating their potential in tendon repair and regeneration under *in vivo* conditions, such as the repair of collagenase-induced tendon injury in animal models.

## 5. Conclusion

In summary, our results demonstrated that forced reprogramming of tenocytes by Yamanaka factors repressed the lineage-specific transcriptional network in the resulting teno-iPSCs, which could be re-activated to some extent by mechanical stretching and ectopic expression of tenogenic transcription factor MKX. Our data suggest that tenocyte-derived iPSCs can be a promising cell source for basic and clinic research on tendon repair.

## Supplementary Material

1

2

3

4

5

6

7

8

9

10

## Abbreviations:

BMSC: bone marrow-derived mesenchymal stem cells
ECM: extracellular matrix
ESC: embryonic stem cell
FCS: fetal calf serum
MEF: mouse embryonic fibroblasts
RT-PCR: reverse transcription PCR
qPCR: quantitative real time-polymerase chain reaction
teno-iPSC: tenocyte-derived induced pluripotent stem cells
TPSC: tendon progenitor/stem cell
