# Supplementary figures and images for: Regulation of the tenogenic gene expression in equine tenocyte-derived induced pluripotent stem cells by mechanical loading and Mohawk
Source: Stem Cell Res. Author manuscript; Available in PMC 2020 Mar 20. (PMC7082636; doi:10.1016/j.scr.2019.101489)

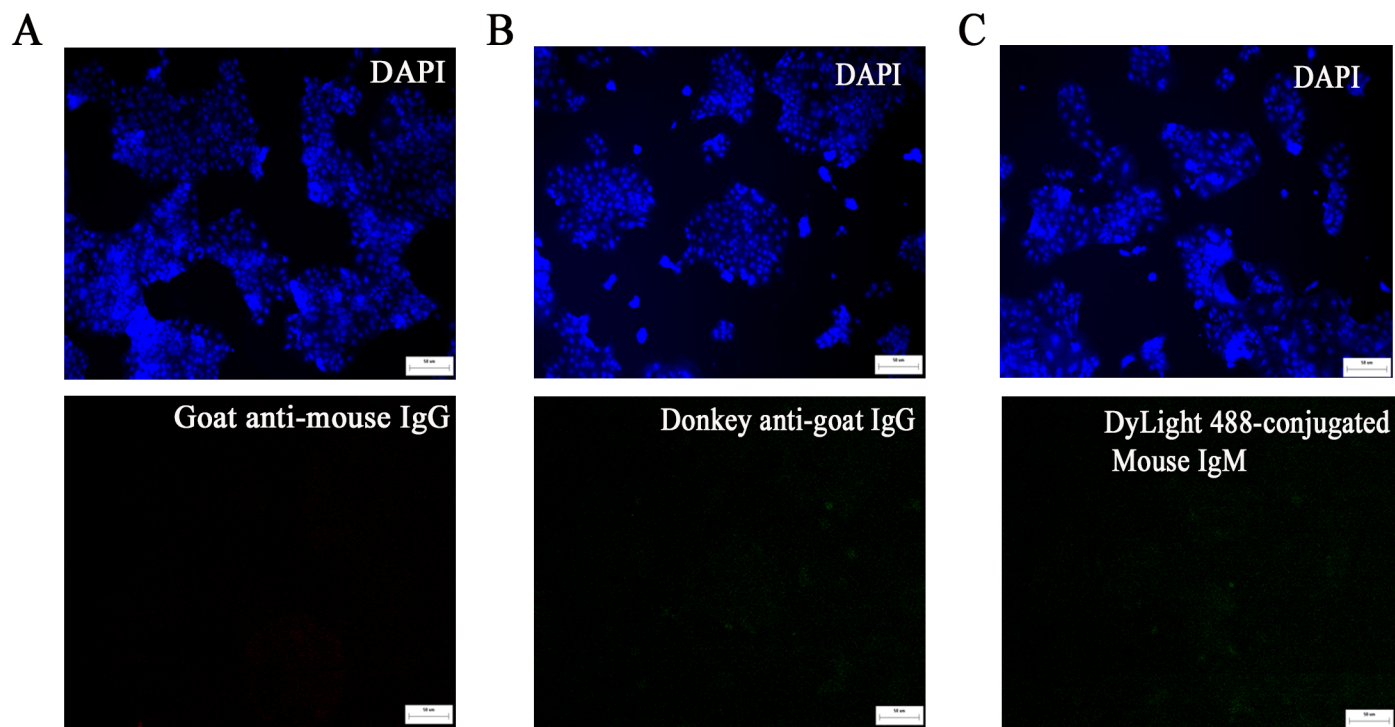

Supplement: 1 [file NIHMS1539478-supplement-1.pdf]

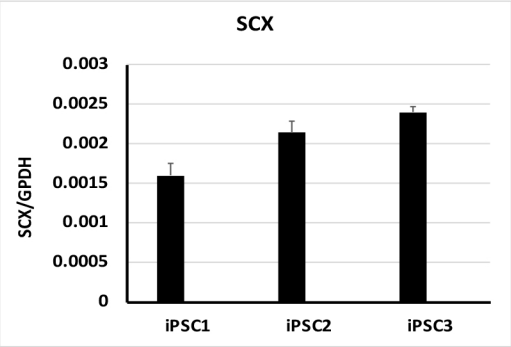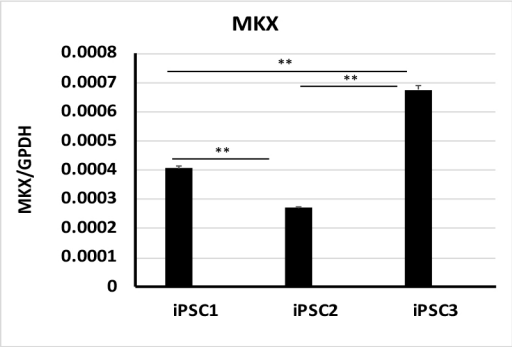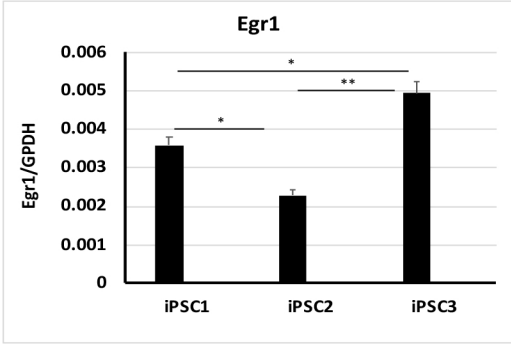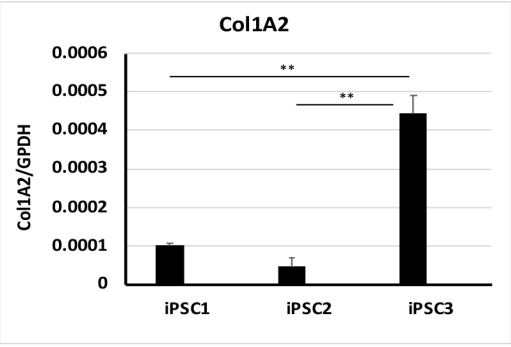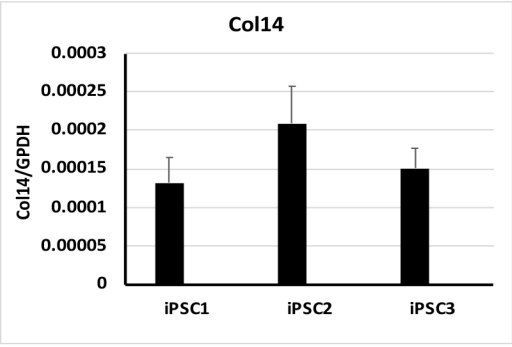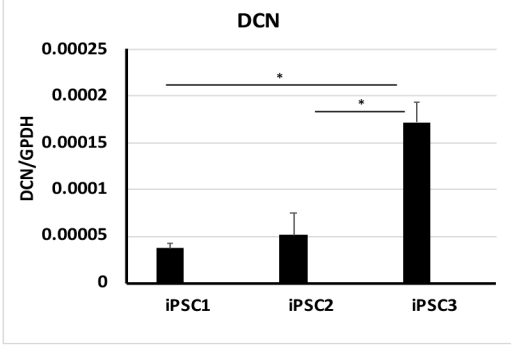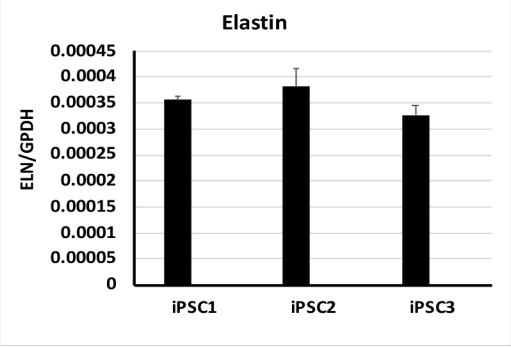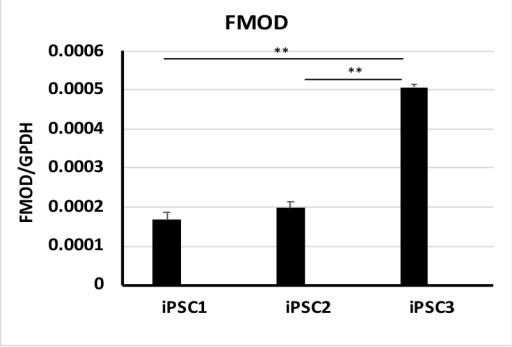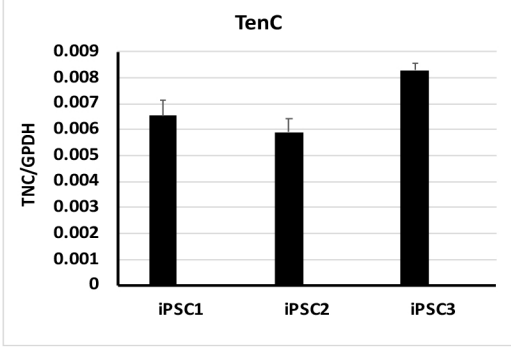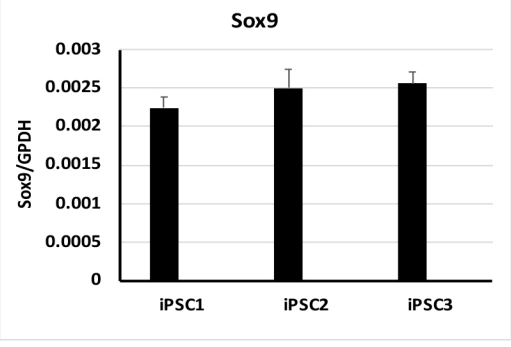

Supplement: 3 [file NIHMS1539478-supplement-3.pdf]

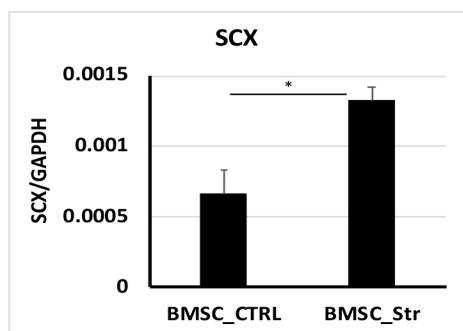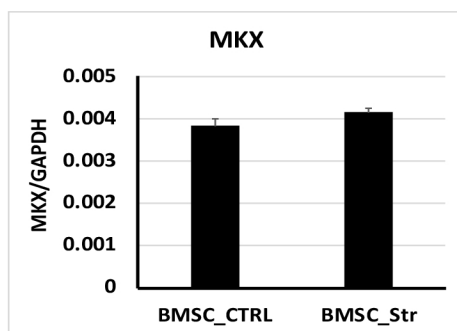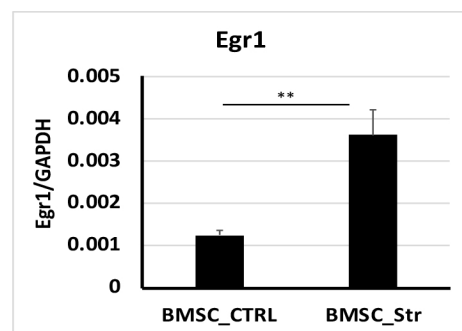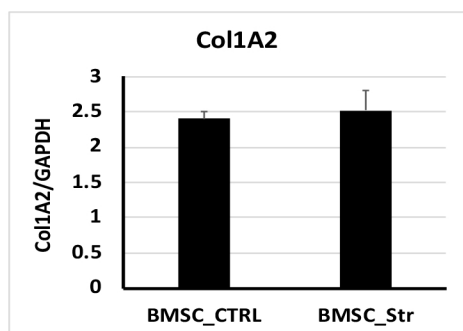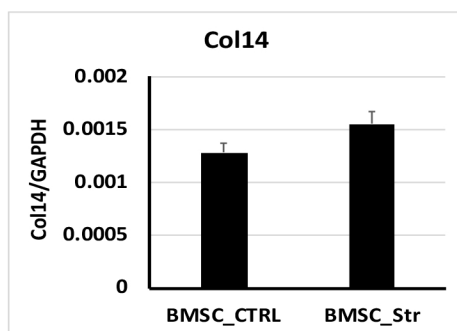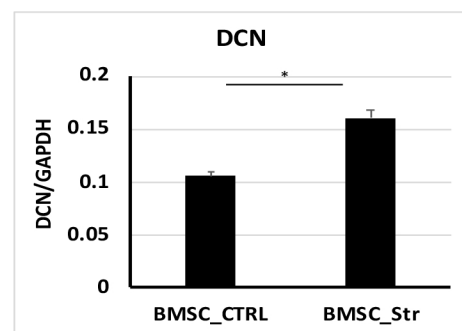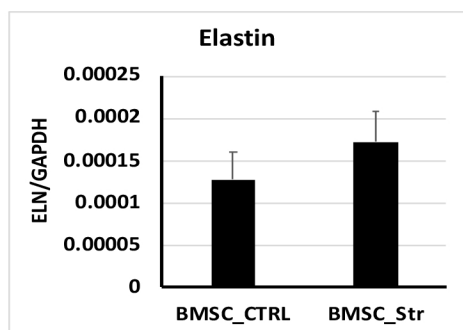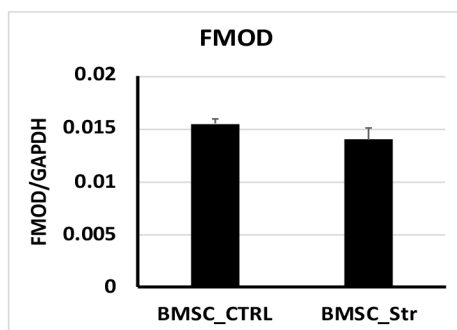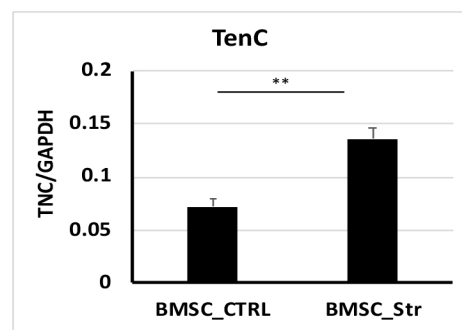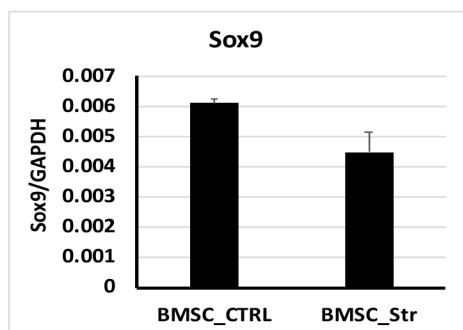

Supplement: 4 [file NIHMS1539478-supplement-4.pdf]

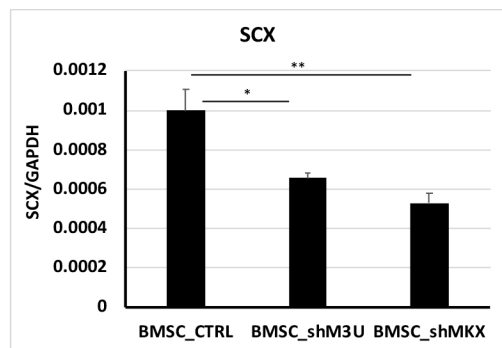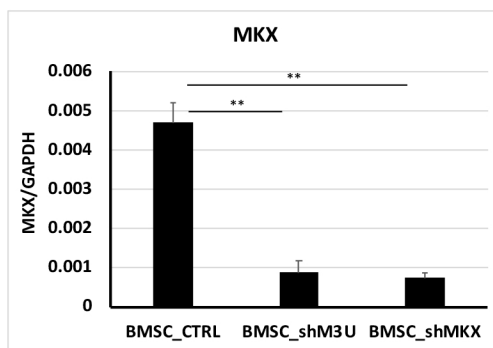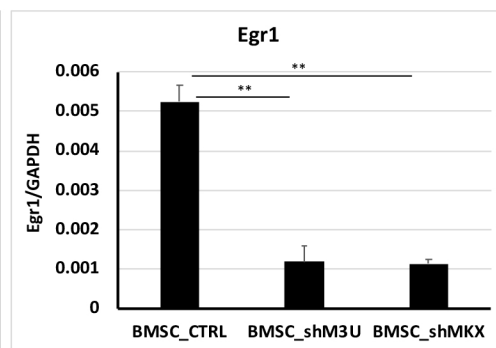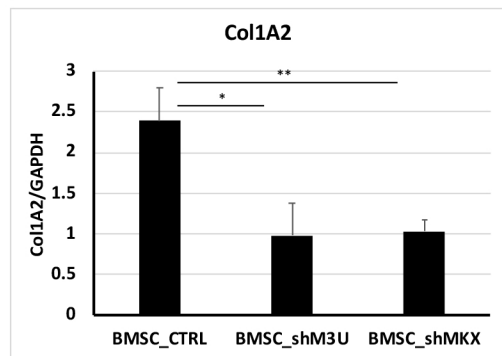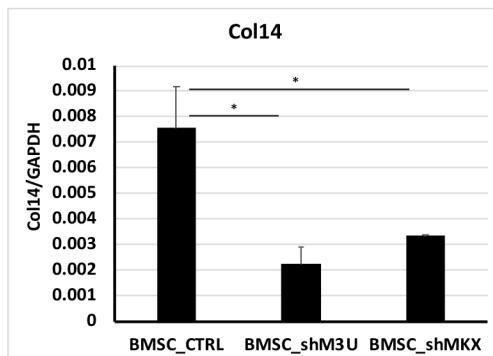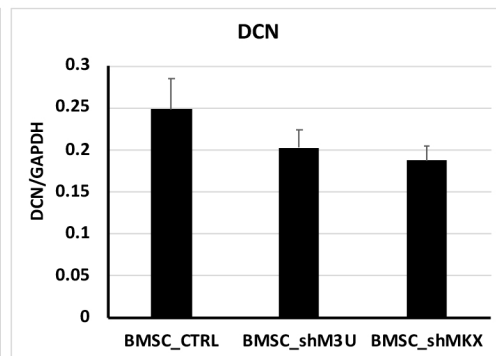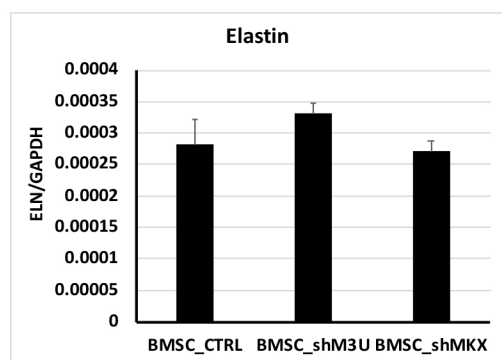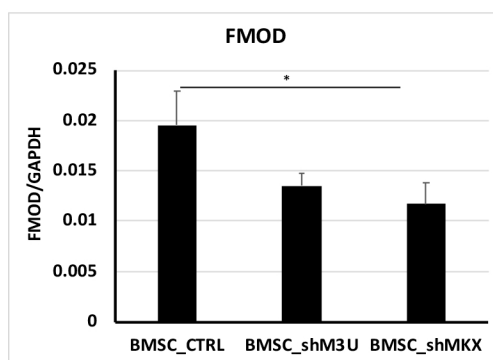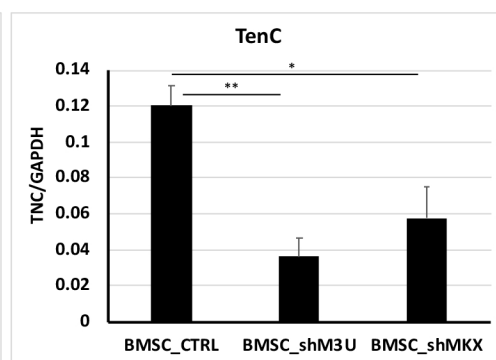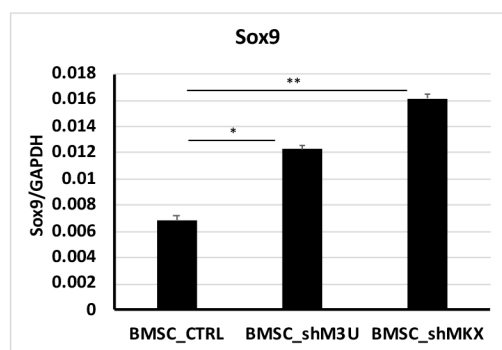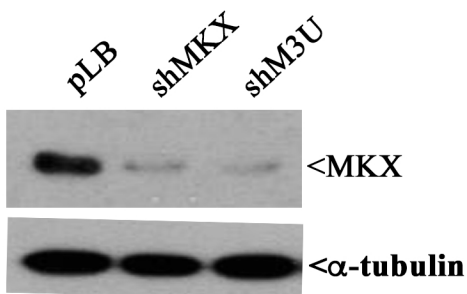

Supplement: 5 [file NIHMS1539478-supplement-5.pdf]

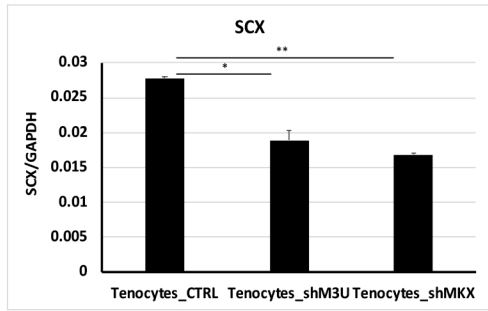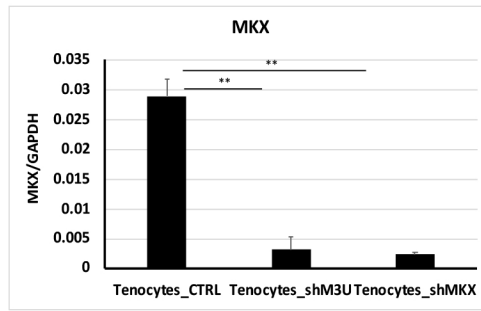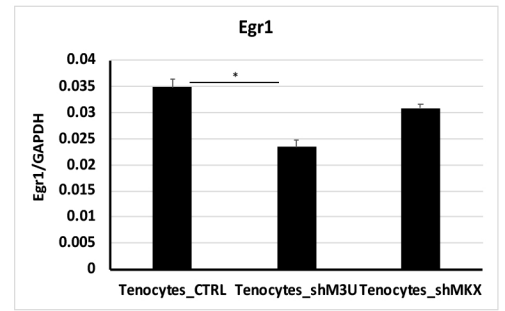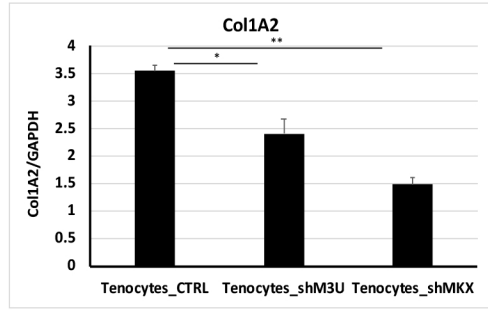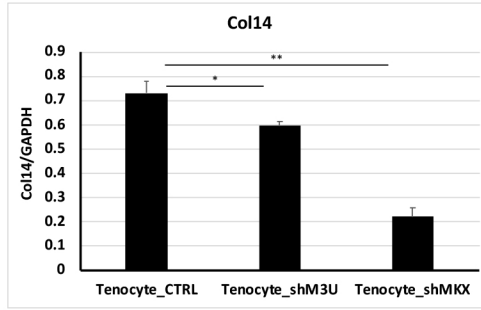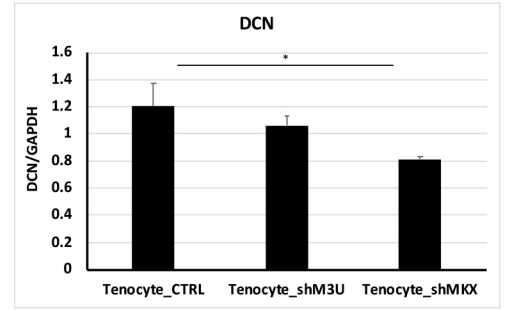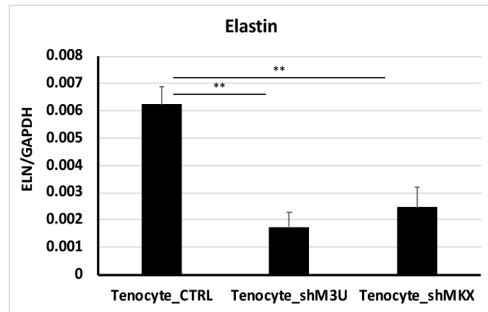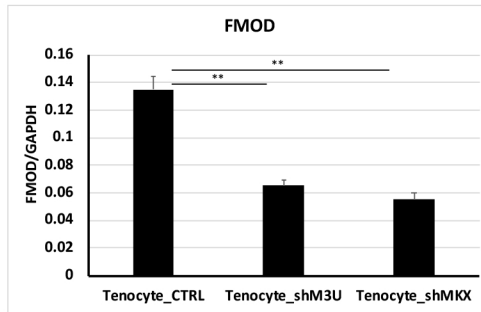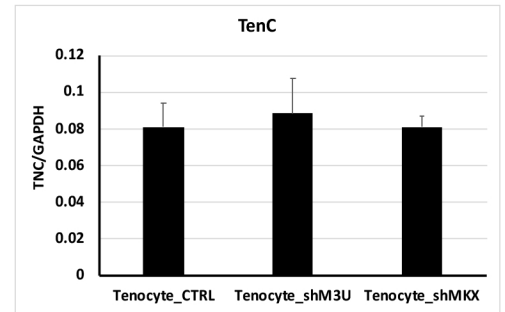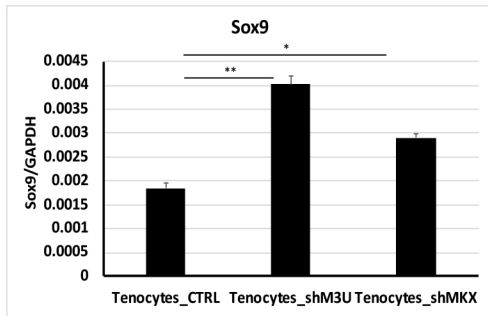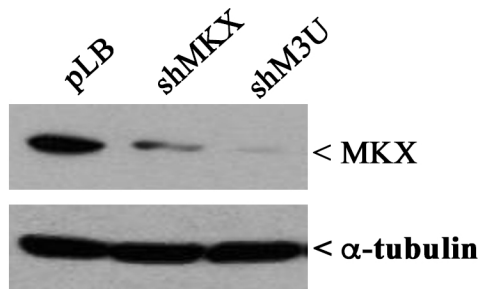

Supplement: 6 [file NIHMS1539478-supplement-6.pdf]

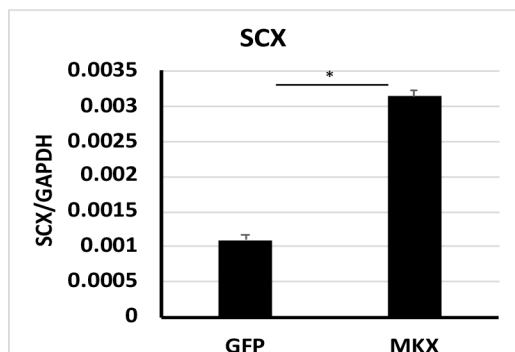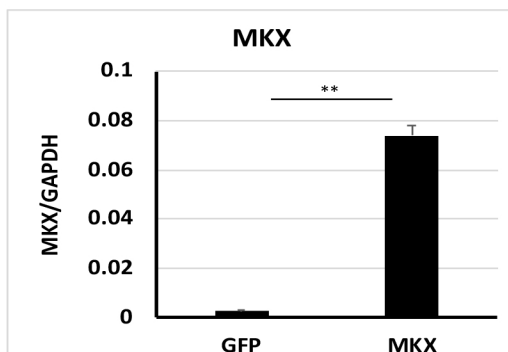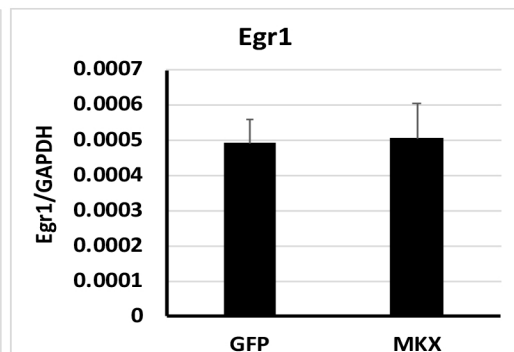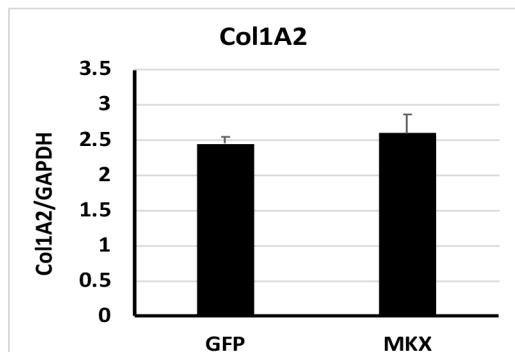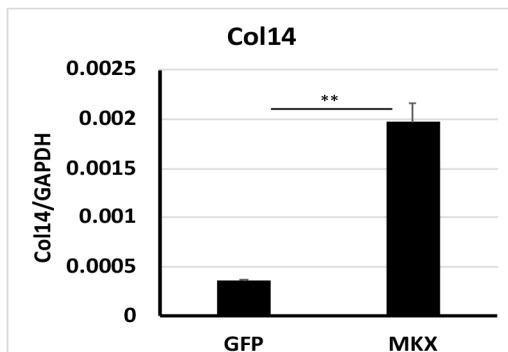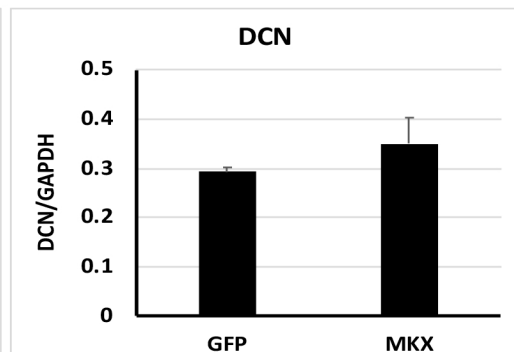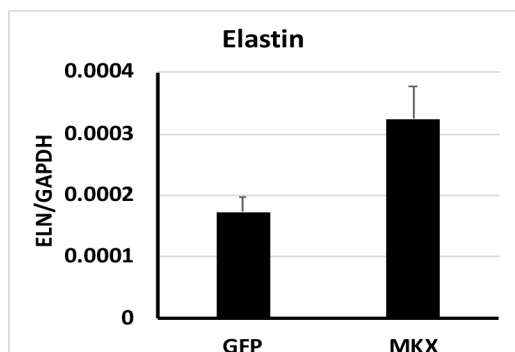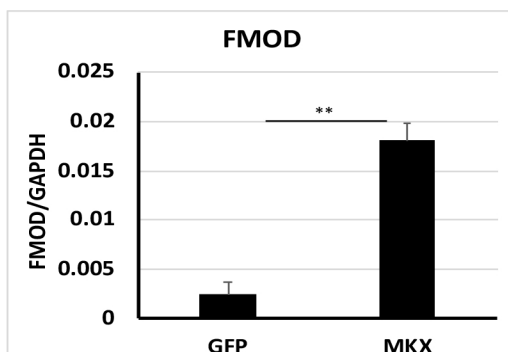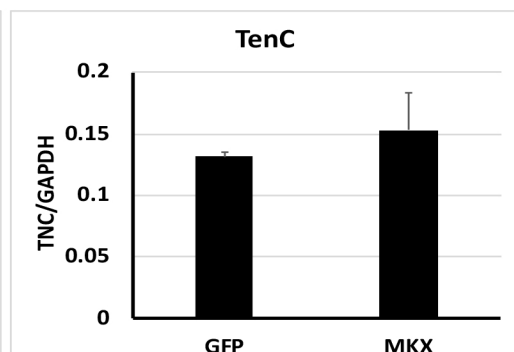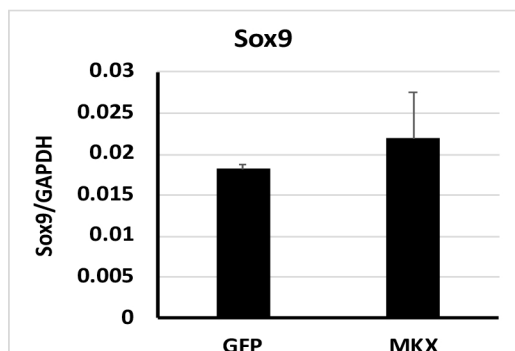

Supplement: 7 [file NIHMS1539478-supplement-7.pdf]

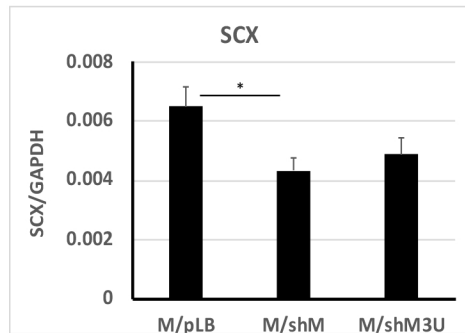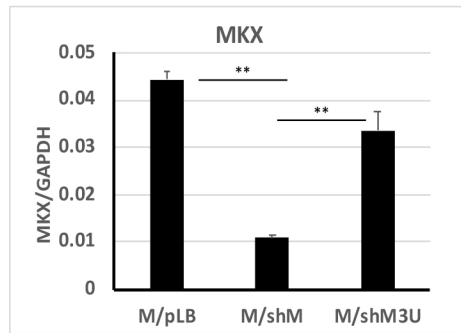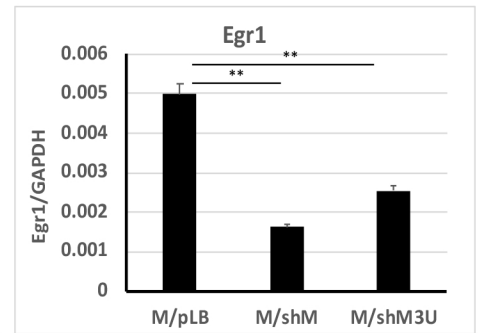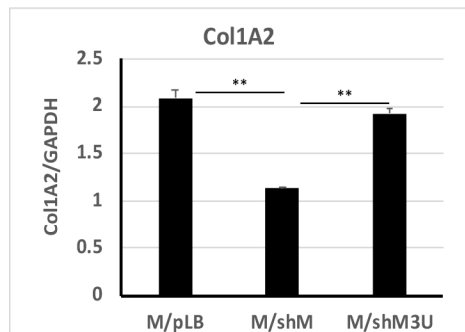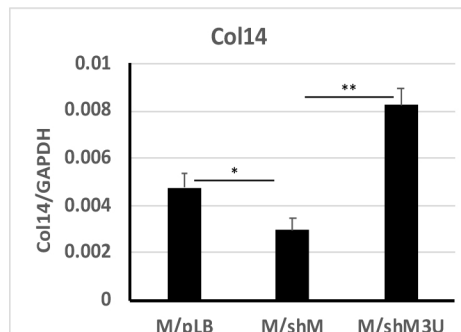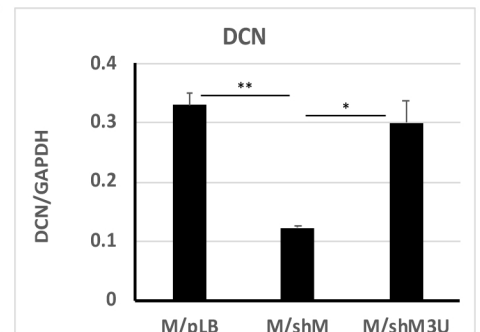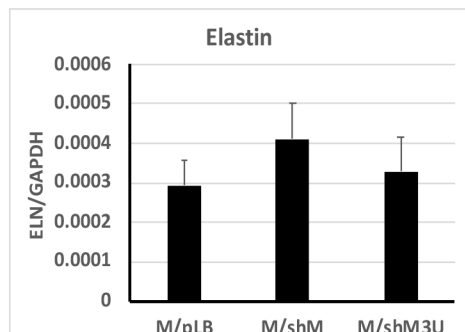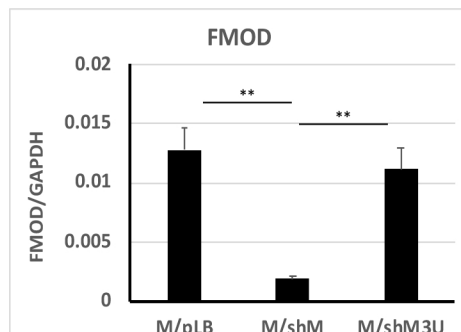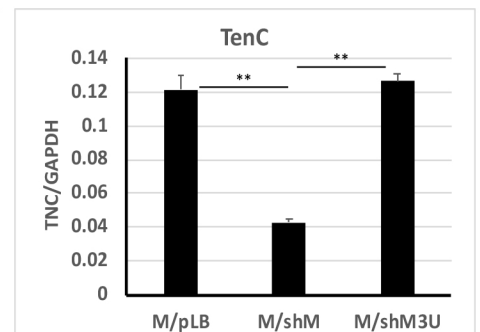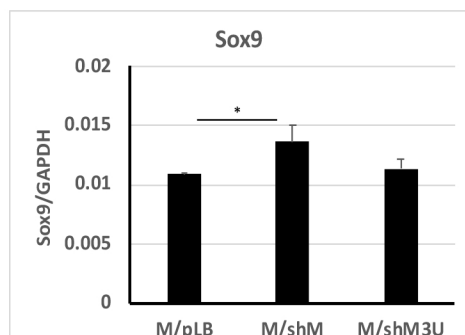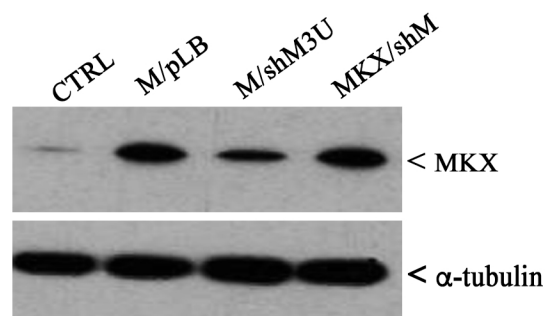

Supplement: 8 [file NIHMS1539478-supplement-8.pdf]

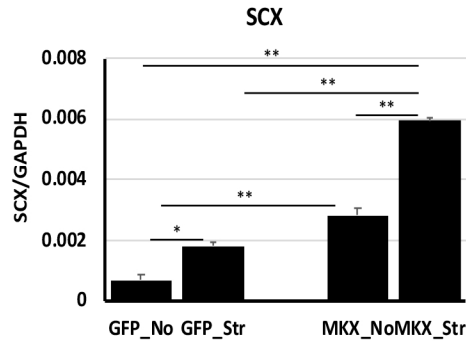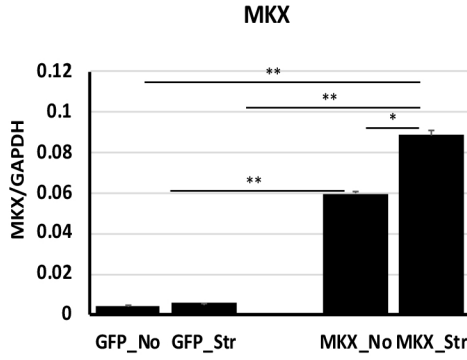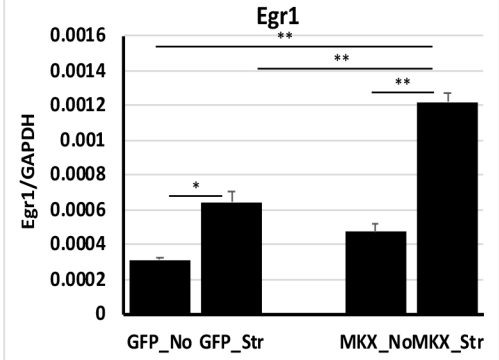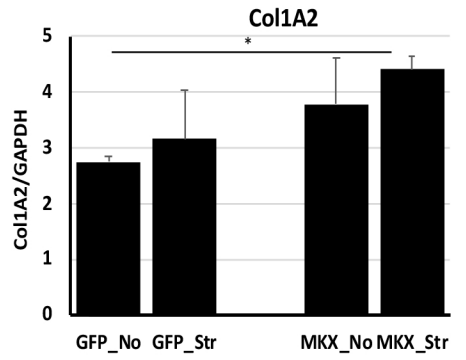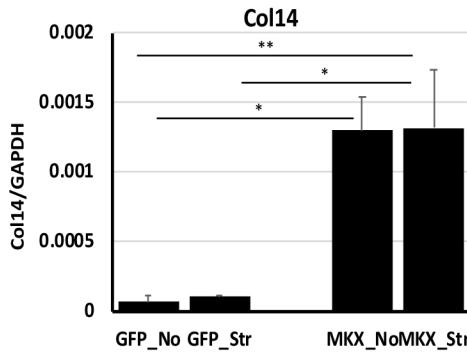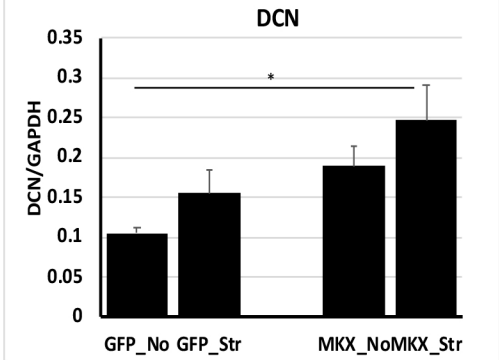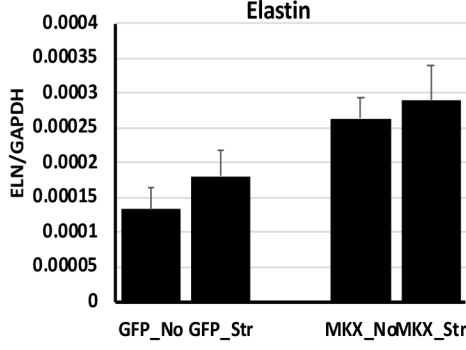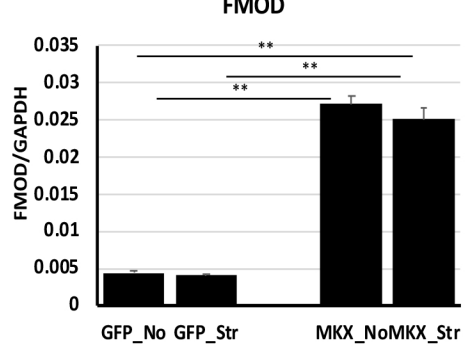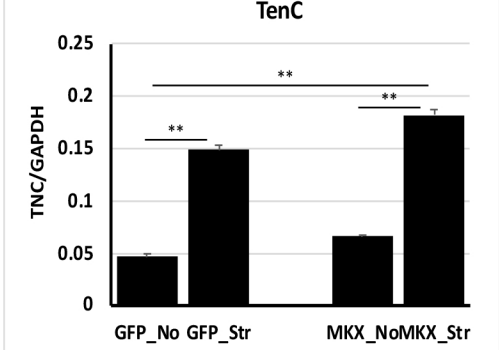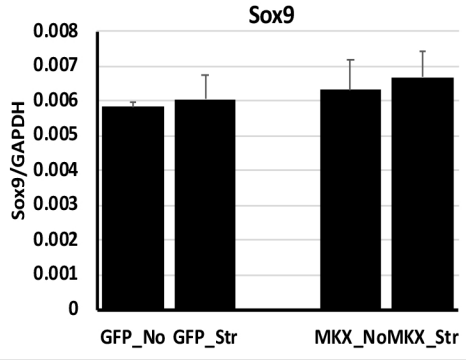

Supplement: 9 [file NIHMS1539478-supplement-9.pdf]
