# Supplementary material for: Regulation of the tenogenic gene expression in equine tenocyte-derived induced pluripotent stem cells by mechanical loading and Mohawk
Source: Stem Cell Res. Author manuscript; Available in PMC 2020 Mar 20. (PMC7082636; doi:10.1016/j.scr.2019.101489)
Supplement: 2 [file NIHMS1539478-supplement-2.docx]

**Supplemental table 2 shRNA Oligonucleotide sequences**

| Oligo name | Oligo sequences (5’ -> 3’) |
| --- | --- |
| shM3U-top | TGCATGTCTCTTGCCTGCATATTTCAAGAGAATATGCAGGCAAGAGACATGCTTTTTTC |
| shM3U-bottom | TCGAGAAAAAAGCATGTCTCTTGCCTGCATATTCTCTTGAAATATGCAGGCAAGAGACATGCA |
| shMKX-top | TGCAGCGATGGCCTTGACAAATTTCAAGAGAATTTGTCAAGGCCATCGCTGCTTTTTTC |
| shMKX-bottom | TCGAGAAAAAAGCAGCGATGGCCTTGACAAATTCTCTTGAAATTTGTCAAGGCCATCGCTGCA |
