# Supplementary material for: Regulation of the tenogenic gene expression in equine tenocyte-derived induced pluripotent stem cells by mechanical loading and Mohawk
Source: Stem Cell Res. Author manuscript; Available in PMC 2020 Mar 20. (PMC7082636; doi:10.1016/j.scr.2019.101489)
Supplement: 10 [file NIHMS1539478-supplement-10.docx]

Supplemental table 1 List of primers used in this study

| Gene Name | | Sequence (5’ 🡪 3’) | Product size (bp) |
| --- | --- | --- | --- |
| *AFP* | For: | CAACTGTGGGCAATTTACAGCA | 233 |
|  | Rev: | CAACTGTGGGCAATTTACAGCA |  |
| Aggrecan  (*ACAN*) | For: | GCGGTACGAGATCAACTCCC | 163 |
|  | Rev: | GGAGCTCCGCTTCTGTAGTC |  |
| *Col1A2* | For: | CGGGAGGTTTCGGCTAAGTT | 218 |
|  | Rev: | TTCCTGCAGTTGCCTCTTGT |  |
| *Col14A1* | For: | CTGGACGATGGAAGTGAG | 215 |
|  | Rev: | GTGACCCTGAACTGCTGC |  |
| Decorin  (*DCN)* | For: | TTATCAAAGTGCCTGGTG | 204 |
|  | Rev: | CATAGACACATCGGAAGG |  |
| *DNMT3B* | For: | TCTGATTCCAAGGACGCACC | 185 |
|  | Rev: | ACTGGTGTGTCGGAACCATC |  |
| *Egr1* | For: | CCTACGAGCACCTGACCTCAG | 241 |
|  | Rev: | GATGGTGCTGAAGATGAAGTGG |  |
| Elastin (*ELN*) | For: | CTATGGTGTCGGTGTCGGAG | 247 |
|  | Rev: | GGGGGCTAACCCAAACTGAG |  |
| *FMOD* | For: | GCTTCTGCTGAGGGACAC | 90 |
|  | Rev: | GATTTCTGGGGTTGGGAC |  |
| *GAPDH* | For: | GTGTCCCCACCCCTAACG | 131 |
|  | Rev: | AGTGTAGCCCAGGATGCC |  |
| GFPA | For: | GGGGGCAAAAGCACCAATG | 198 |
|  | Rev: | CCGCATCTCGACAGTCTTCA |  |
| Leptin (*LEP*) | For: | GAAGAGAGCCTGTGTGGAC | 244 |
|  | Rev: | TGAAATCATCCCGGGTCAC |  |
| Mohawk  (*Mkx*) | For: | TAATCCCGTTCACCATCC | 195 |
|  | Rev: | CTTTGCCTTGTCTTTCCC |  |
| *Nanog* | For: | TCCTCAATGACAGATTTCAGAGA | 323 |
|  | Rev: | GAGCACCAGGTCTGACTGTTCC |  |
| Nestin  *(NES)* | For: | ACTGAGAAGTTCCAGCTGGC | 158 |
|  | Rev: | TCAGCCTCTAGAAGGGTCC |  |
| Oct4  (*POU5F1*) | For: | GGGACCTCCTAGTGGGTCA | 318 |
|  | Rev: | TGGCAAATTGCTCGAGGTCT |  |
| Osteocalcin  (*BGLAP*) | For: | GTGCAGAGTCTGGCAGAGGT | 93 |
|  | Rev: | CCAGCCAATGATCCAGGTAG |  |
| *Pax6* | For: | GCTGCTATCTGTCCTTGGCT | 187 |
|  | Rev: | CCGAGTTGATTCACTCCGCT |  |
| *REX1* | For: | GACGGGAAAGGCCTGGATAGAAG | 297 |
|  | Rev: | GGCGGTAAGAAGCTGTTGAGAAAGG |  |
| Scleraxis  (*Scx*) | For: | CCCCCACGGACCTGACTC | 167 |
|  | Rev: | GGTAGGAAGCCAGCACGG |  |
| *Sox9* | For: | CTGGAGACTGCTGAACGAGA | 171 |
|  | Rev: | GAGATGTGTGTCTGCTCCGT |  |
| Tenascin C  (*TNC*) | For: | GAACACGGTGGAGTATGC | 105 |
|  | Rev: | TTGGTAGTGATGGCTGAG |  |
| *TNMD#1* | For: | GGCGGGTTATCTGTCGTG | 169 |
|  | Rev: | TACCAGGAGCCAAATGCC |  |
| *TNMD#2* | For: | GGTCCCAGCAGAAAAGCCTAT | 316 |
|  | Rev: | CTCATCCAGCATGGGGTCAA |  |
| *TNMD#3* | For: | GGTCCCAGCAGAAAAGCCTA | 128 |
|  | Rev: | AGTCTTGTAACTCTGAAACTGCT |  |
